# Deep-trap ultraviolet persistent phosphor for advanced optical storage application in bright environments

**DOI:** 10.1038/s41377-024-01533-y

**Published:** 2024-09-14

**Authors:** Xulong Lv, Yanjie Liang, Yi Zhang, Dongxun Chen, Xihui Shan, Xiao-Jun Wang

**Affiliations:** 1 Key Laboratory for Liquid-Solid Structure Evolution and Processing of Materials, Shandong University, Jinan, 250061 China; 2 Department of Physics, Georgia Southern University, Statesboro, GA 30460 USA

**Keywords:** Optical materials and structures, Inorganic LEDs

## Abstract

Extensive research has been conducted on visible-light and longer-wavelength infrared-light storage phosphors, which are utilized as promising rewritable memory media for optical information storage applications in dark environments. However, storage phosphors emitting in the deep ultraviolet spectral region (200–300 nm) are relatively lacking. Here, we report an appealing deep-trap ultraviolet storage phosphor, ScBO_3_:Bi^3+^, which exhibits an ultra-narrowband light emission centered at 299 nm with a full width at half maximum (FWHM) of 0.21 eV and excellent X-ray energy storage capabilities. When persistently stimulated by longer-wavelength white/NIR light or heated at elevated temperatures, ScBO_3_:Bi^3+^ phosphor exhibits intense and long-lasting ultraviolet luminescence due to the interplay between defect levels and external stimulus, while the natural decay in the dark at room temperature is extremely weak after X-ray irradiation. The impact of the spectral distribution and illuminance of ambient light and ambient temperature on ultraviolet light emission has been studied by comprehensive experimental and theoretical investigations, which elucidate that both O vacancy and Sc interstitial serve as deep electron traps for enhanced and prolonged ultraviolet luminescence upon continuous optical or thermal stimulation. Based on the unique spectral features and trap distribution in ScBO_3_:Bi^3+^ phosphor, controllable optical information read-out is demonstrated via external light or heat manipulation, highlighting the great potential of ScBO_3_:Bi^3+^ phosphor for advanced optical storage application in bright environments.

## Abstract (web summary)

We report a deep-trap ultraviolet persistent phosphor with thermoluminescence glow peaks beyond 500 K that exhibits intense and long-lasting ultraviolet luminescence under indoor lighting conditions but emits negligible afterglow in darkness.

## Graphical abstract

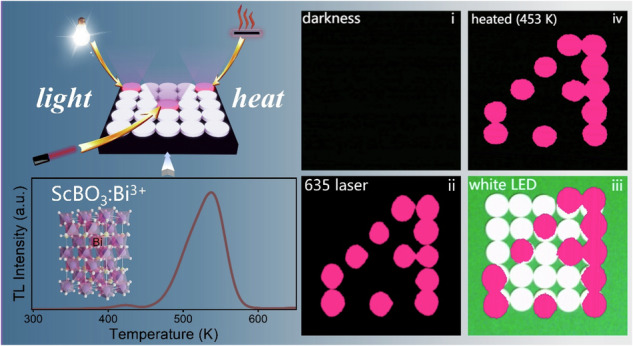

## Introduction

As human society enters the 21st century, commonly called the era of “big data”, the volume of digital data is experiencing exponential growth. It is estimated that the total amount of digital data will surpass 1011 TB by 2025^1,2^. The explosive growth of digital data has raised significant concerns worldwide regarding information storage and encryption^3–5^. Currently, among the various information storage techniques, semiconductor, magnetic, and optical-based methods are the primary ones^6,7^. Optical information storage, in particular, holds great promise due to its numerous unparalleled advantages, including long lifetime, fast speed, low energy consumption, and easy portability^8–10^. Storage phosphors, which can store excitation energy and release it as light emission upon external thermal, optical, or other physical stimulations, have been extensively studied and applied as rewritable optical memory media for optical information storage in recent years^11–15^. In contrast to those thermally stimulated persistent luminescence (PersL) materials at room temperature, photo-stimulated luminescence (PSL) materials ensure light emission when the pre-irradiated phosphors are exposed to external light stimulus, enabling long-term energy storage before photo-stimulation at room temperature^16–19^. The distinctive capability of PSL materials to absorb, store, and release energy on-demand has sparked extensive research and application of these storage phosphors in various critical fields, such as dosimetry, computed radiography, and optical information storage^20–22^.

The past decades have witnessed the significant progress in the development of visible-light and longer-wavelength infrared-light PSL materials, including silicates (e.g., Ba_2_SiO_4_:Eu^2+^, Ho^3+^)^23^, halides (e.g., BaFBr:Eu^2+^)^24^, nitrides (e.g., CaAlSiN_3_:Eu^2+^)^25^, gallates (e.g., LiGa_5_O_8_:Cr^3+^)^26^, and others^27–29^, which perform well for the application of optical information storage. For example, Liu et al. proposed a PSL material through controllable co-doping of Dy^3+^, Tm^3+^, and Ho^3+^ into green-emitting Ba_2_SiO_4_:Eu^2+^ phosphor for multilevel information storage under external optical or thermal stimulation^27^. Zhuang et al. reported a series of deep-trap oxynitride storage phosphors (Sr_1-x_Ba_x_)Si_2_O_2_N_2_:Eu/Yb, Dy (490–620 nm), demonstrating multidimensional optical information storage via emission intensity/wavelength multiplexing^2^. Lin et al. developed a transparent glass-ceramic infused with LiGa_5_O_8_:Mn^2+^ nanocrystals (510 nm and 625 nm), in which the information within remains visually imperceptible until subjected to specific conditions: either heating to temperature exceeding 423 K or exposure to near-infrared (NIR) light irradiation^28^. However, it should be noted that ambient light typically falls within the visible and NIR spectral ranges, which can partially affect or overshadow the visible or NIR luminescence signals from these storage phosphors, that is, these phosphors can only be used for optical information storage in dark environments to avoid the interference of ambient light^30–33^. To enable information encryption and storage in ambient lighting conditions, it is imperative to completely separate the luminescence signals from the ambient light spectrum. In this case, storage phosphors emitting in the deep ultraviolet region are preferred, considering that deep ultraviolet radiation encompassing the light spectrum over 200–300 nm, does not overlap with room light and can be detected with zero background noise in a bright indoor-lighting environment^34–38^. Furthermore, optical data storage application usually requires the storage phosphors to have a large trap depth (usually >1 eV) and high trap density to ensure storage efficiency in dark environments and high PSL efficiency upon external light stimulation. Nevertheless, deep-trap storage phosphors emitting in the shorter-wavelength deep ultraviolet spectral region are relatively scarce.

As a favorable UV-emitting center, trivalent bismuth ion (Bi^3+^) has garnered significant research interest due to its 6s6p→6s^2^ allowed transitions^39–41^. With an electronic configuration of [Xe]4f^14^5d^10^6s^2^, the exposed 6s electrons of Bi^3+^ exhibit high sensitivity to the crystal field of the surrounding environment. Consequently, the energy level positions of Bi^3+^ vary significantly in different host matrices, resulting in a broad range of emissions spanning from deep ultraviolet to NIR spectral regions^42–45^. However, most Bi^3+^ ion doped ultraviolet-emitting persistent phosphors were concentrated on the ultraviolet-A spectral range (*λ* ≥ 320 nm) such as Sr_3_Y_2_Ge_3_O_12_:Bi^3+^ (354 nm)^46^, LiScGeO_4_:Bi^3+^ (360 nm)^47,48^, CdSiO_3_:Bi^3+^ (360 nm)^49^, SrLaAlO_4_:Bi^3+^ (380 nm)^50^, and others^51,52^. Although deep-trap persistent phosphors emitting at shorter-wavelength deep ultraviolet regions hold great potential for detectable and interference-free light emissions in bright environments, it is noteworthy that, to the best of our knowledge, Bi^3+^-doped deep-trap storage phosphors with central emission peaks below 300 nm have rarely been reported. On the other hand, to realize deep ultraviolet luminescence of Bi^3+^, the host materials with large band gap and weak crystal fields are needed^43^ since the emissions of Bi^3+^ are closely related to the host environment.

In this work, we report a novel deep-trap ultraviolet storage phosphor ScBO_3_:Bi^3+^, which shows a remarkably narrowband ultraviolet emission centered at 299 nm with an unprecedented FWHM of approximately 0.21 eV, along with exceptional capabilities for storing X-ray energy. The effects of the spectral distribution of ambient light and ambient temperature on ultraviolet light emission have been investigated in detail. Notably, long-lasting ultraviolet light emission is realized through the continuous photo-stimulation of longer-wavelength white light/NIR light or thermal stimulation at elevated temperatures. Besides, comprehensive spectroscopic analyses and first principles investigations have been conducted to clarify the nature of energy traps and the ultraviolet luminescence mechanisms in this ScBO_3_:Bi^3+^ phosphor. This work provides valuable insights and perspectives for the design of deep-trap ultraviolet storage phosphors.

## Results

### Crystal structure and narrowband ultraviolet photoluminescence

The crystal structure, morphology, and element distribution of the as-synthesized ScBO_3_:Bi^3+^ compound were studied by X-ray diffraction, scanning electron microscopy, and dispersive spectroscopy spectrum, as shown in Fig. S1 and Table S1. The corresponding results suggest that ScBO_3_:Bi^3+^ is of a single phase with the elements evenly distributed within a randomly selected particle.

**Fig. 1:**
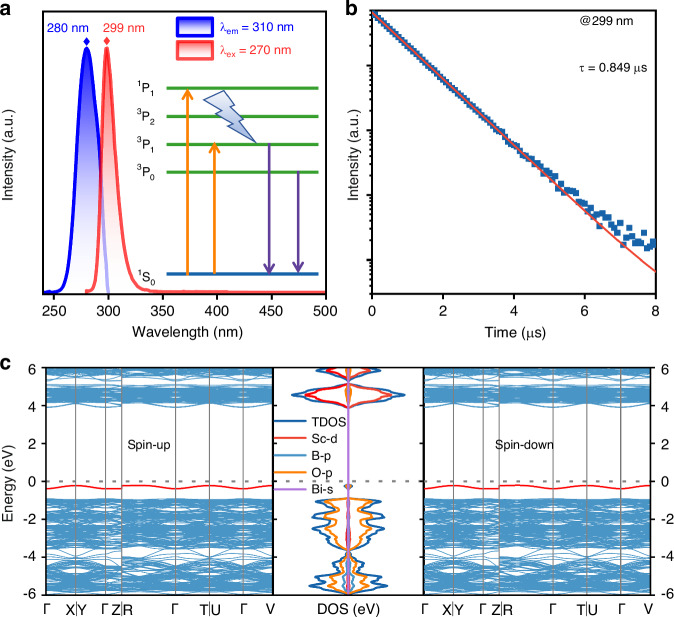
Ultraviolet photoluminescence and electronic band structure. **a** Room temperature photoluminescence excitation and emission spectra of the ScBO_3_:Bi^3+^ phosphor. The emission spectrum is obtained under 270 nm light excitation, while the excitation spectrum is acquired by monitoring at 310 nm. The inset shows the energy level scheme of the Bi^3+^ ion and the possible optical transitions. **b** The room temperature luminescence decay curve and corresponding single exponential fitting curve of the ScBO_3_:Bi^3+^ phosphor monitored at 299 nm. **c** Electronic band structure and DOS of the ScBO_3_:Bi^3+^ phosphor. The red solid line indicates the newly created electron energy level after the introduction of Bi^3+^

Figure 1a shows the normalized room temperature photoluminescence emission and excitation spectra of the ScBO_3_:Bi^3+^ phosphor. Inset is the energy level structure of the Bi^3+^ ion. When excited with 270 nm light, the ScBO_3_:Bi^3+^ phosphor exhibits an ultra-narrow emission band in the ultraviolet spectral range with a peak maximum at 299 nm and a FWHM of 0.21 eV (Fig. S2a), which can be attributed to the ^3^P_0,1_ → ^1^S_0_ transition of Bi^3+^. Phosphors with various Bi doping concentrations exhibit similar emission spectra that display an almost identical profile (Fig. S2b). The excitation spectrum monitored at 310 nm emission comprises a narrow absorption band spanning from 255 to 300 nm, with a peak maximum at 280 nm, which can be assigned to the Bi^3+ 1^S_0_ → ^3^P_1_ allowed transition^43^. Figure 1b shows the room-temperature luminescence decay curve monitored at 299 nm, which can be fitted well with a single exponential function:

1 $${I}_{t}={I}_{0}\exp (-t/{\tau })$$

where *I*_*t*_ is emission intensity at time *t*, *I*_0_ is the initial intensity, and *τ* is the lifetime. The lifetime (*τ*) at room temperature is determined to be 0.849 μs. The short lifetime further demonstrates that the emission mainly originates from ^3^P_1_ excited state. To further investigate the transition of Bi^3+^ emitters, the photoluminescence emission and excitation spectra as well as the luminescence decay were also measured at 77 K, as shown in Fig. S3. Excitation with 280 nm light at 77 K produces ultraviolet emissions peaking at 303, 305, 308, and 311 nm, respectively, exhibiting a red shift compared with the room temperature emission. Furthermore, the lifetime (*τ*) at 77 K is measured to be 0.607 ms, which is much longer than that at room temperature. The low-temperature spectral results illustrate that the ultraviolet emission at 77 K is mainly ascribed to the dominant ^3^P_0_ → ^1^S_0_ transition of Bi^3+^^45^.

Given that the density of states (DOS) and electronic band structure will affect the photoluminescence and PSL properties, density functional theory (DFT) calculations were performed. The band structure of the perfect ScBO_3_ host features a direct band gap at the G point with an energy of 4.82 eV, suggesting that the ScBO_3_ host is suitable to accommodate the transitions of Bi^3+^ ions, providing a sufficient energy gap for inducing ultraviolet luminescence (Fig. S4a). Moreover, it is found that the Sc atoms mainly form the conduction band (CB), while the valence band (VB) is derived from the B atoms and O atoms. The partial DOS discloses that the Sc 3d orbital sets the bottom of CB, while the O 2p states dominate the top of VB (Fig. S4b). The introduction of Bi^3+^ modifies the local electronic structures. It forms the 6 s level of Bi and the 2p level of O in the band gap, as shown by the red solid line in Fig. 1c. Accordingly, the changed electronic band structure and newly formed electron level may facilitate the formation of energy traps, enabling long-lasting ultraviolet luminescence of the ScBO_3_:Bi^3+^ storage phosphor in bright environments.

### Indoor white light stimulated ultraviolet luminescence in ScBO_3_:Bi^3+^ phosphor

Besides the ultra-narrowband ultraviolet photoluminescence, the ScBO_3_:Bi^3+^ phosphor also exhibits intense ultraviolet light emission in the bright indoor-lighting environment after ceasing high-energy X-ray irradiation, although the natural decay in the dark is extremely weak. This can be attributed to the fact that most trapped electrons are stored in deep energy traps of ScBO_3_:Bi^3+^ phosphor that cannot be released efficiently by room-temperature thermal stimulation. The thermoluminescence (TL) glow curve of the pre-irradiated ScBO_3_:Bi^3+^ phosphor further proves this, as depicted in Fig. S5. According to the Gaussian fitting results, there are four TL emission peaks at 370 K (Trap I), 423 K (Trap II), 522 K (Trap III), and 547 K (Trap IV), respectively, and the deep traps at high temperature (522 K and 547 K) dominate the TL spectrum. Furthermore, the TL glow curves of the ScBO_3_:Bi^3+^ phosphors with varied Bi^3+^ doping concentrations and excitation duration were also recorded. As presented in Fig. S6, the optimal doping concentration is determined to be 0.1% and 25 min of X-ray excitation can fully charge the ScBO_3_:Bi^3+^ phosphor.

**Fig. 2:**
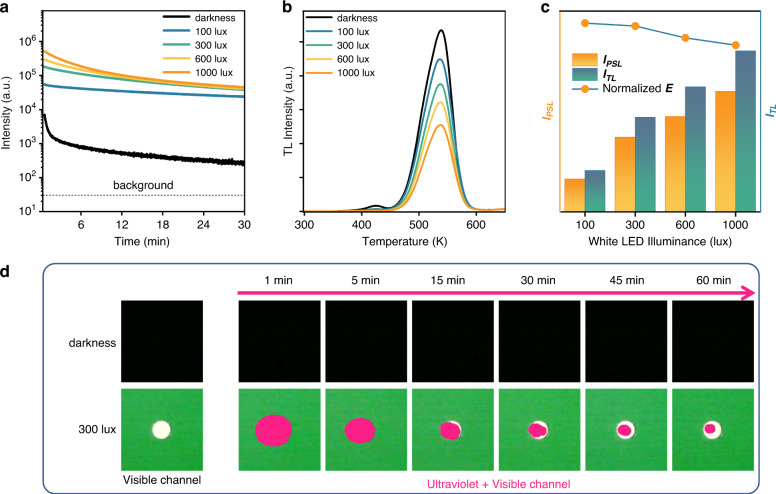
Ultraviolet PersL in dark and bright environments. **a** Ultraviolet PersL decay curves monitored at 299 nm under different illuminance (0, 100, 300, 600, 1000 lux) after irradiation by X-ray. **b** TL curves of the ScBO_3_:Bi^3+^ phosphor after 30 min decay under different illuminance (0, 100, 300, 600, 1000 lux). The ScBO_3_:Bi^3+^ phosphor was first irradiated by X-ray for 25 min in the dark and immediately transferred to the spectrometer for testing, in which different white light illuminances were applied. After 30 min decay, the TL glow curves of the phosphors were measured. **c** The integral of the enhanced ultraviolet luminescence intensity (*I*_*PSL*_) and the decreased TL intensity (*I*_*TL*_) along with the normalized effectiveness factor (*E*) of the ScBO_3_:Bi^3+^ phosphor under white LED illumination (100, 300, 600, and 1000 lux). The effectiveness factor (*E*) is defined as *E* = *I*_*PSL*_/*I*_*TL*_. **d** Ultraviolet luminescence images of the ScBO_3_:Bi^3+^ phosphor disc recorded by the ultraviolet camera upon darkness and 300 lux white LED light illumination at room temperature

Figure 2a shows the ultraviolet PersL decay curves of the ScBO_3_:Bi^3+^ phosphor monitored at 299 nm at room temperature under different illuminance (0, 100, 300, 600, 1000 lux). Compared with the natural decay in the dark (black solid line), the ultraviolet emission intensity of the ScBO_3_:Bi^3+^ phosphor realizes a significant enhancement under the illumination of white LED light. Moreover, the ultraviolet PersL decay curve is affected by the applied illuminance of white light, the ultraviolet afterglow decay curves with higher initial emission intensity and faster decay rate were obtained when exposed to white light with higher illuminance, indicating the accelerated release of electrons in the deep traps under white LED illumination. This is also confirmed by the significant decrease of the TL intensity compared to the naturally decayed phosphor in the dark, as depicted in Fig. 2b. With increasing the illuminance of indoor white light, the decrease of the TL intensity becomes more pronounced, illustrating the interplay between white light and deep traps produces an enhanced ultraviolet light emission. And white light with higher illuminance prefer to facilitate the release of more stored electrons. However, it is unknown whether the released energy is completely utilized to recombine with Bi^3+^ emitting centers for enhanced ultraviolet luminescence. To better demonstrate the effectiveness of polychromatic white light photo-stimulation, the effectiveness factor (*E*) is introduced, which establishes a link between the decrease in TL intensity and the enhancement in ultraviolet luminescence intensity in a certain period and can reflect the overall release efficiency of the stored energy in the phosphor. Considering the decreased TL intensity as the “input” and the enhanced luminescence intensity as the “output”, the effectiveness factor is given by:

2 $$E={I}_{{PSL}}/{I}_{{TL}}$$

where *I*_*PSL*_ and *I*_*TL*_ correspond to the integral of the enhanced luminescence intensity and the decreased TL intensity compared to the PersL and TL intensity of the natural decay in the dark. Figure 2c shows the trend of the normalized effectiveness factor (*E*), a larger *E* value means that more de-trapped electrons are used for ultraviolet light emission in bright environments, indicating higher stimulation effectiveness. As the illuminance increases, both *I*_*TL*_ and *I*_*PSL*_ become more pronounced, indicating more deep-trapped electrons were released. However, the normalized effectiveness factor (*E*) gradually decreases as the illuminance increases from 100 to 1000 lux, illustrating that more energy loss occurs upon higher illuminance, and the optimal illuminance of white light is determined to be 100 lux.

Furthermore, the white light stimulated ultraviolet light emission were also visually recorded using the ultraviolet camera at room temperature (Fig. 2d). Each image was recorded by overlaying an ultraviolet image onto a visible one. The ultraviolet luminescence signal, detected and represented by the pink color, corresponds proportionally to the intensity of ultraviolet luminescence. As depicted in Fig. 2d, the ultraviolet emission intensity of the ScBO_3_:Bi^3+^ phosphor in the dark is extremely weak so that it cannot be detected by the camera even at 1 min decay after ceasing X-ray excitation. Nevertheless, when the indoor white LED light is turned on, the ultraviolet luminescence signal is significantly enhanced and becomes observable through the ultraviolet camera. As the illumination time increases, the area of the pink pattern gradually diminishes, indicating a decrease of the ultraviolet persistent luminescence intensity.

**Fig. 3:**
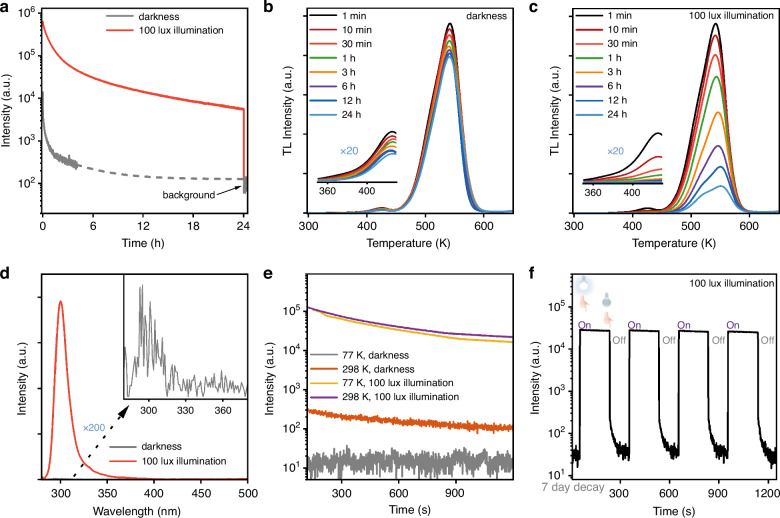
Long persistent ultraviolet luminescence and thermoluminescence in dark and bright environments. **a** Long persistent ultraviolet luminescence decay curves of the ScBO_3_:Bi^3+^ phosphor upon darkness and 100 lux white LED illumination after irradiation by X-ray. **b, c** Time-dependent TL curves of the ScBO_3_:Bi^3+^ phosphor upon darkness and 100 lux white LED light illumination. **d** The emission spectra acquired at 1 h decay upon darkness and 100 lux white LED illumination. **e** Ultraviolet PersL decay curves upon darkness and 100 lux white LED illumination at 77 K and room temperature after irradiation by X-ray at room temperature. **f** White LED light stimulated ultraviolet luminescence of the ScBO_3_:Bi^3+^ phosphor after 7-day natural decay, with 3 min illumination and 2 min natural decay for each cycle

Figure 3a depicts the 24 h natural decay curve in the dark and decay curve under 100 lux white light illumination monitored at 299 nm at room temperature after X-ray irradiation. The data were collected as a function of the emission intensity at 299 nm against decay time. For the natural decay curve in the dark, as the gray line shows, the ultraviolet luminescence intensity is extremely weak and declines very quickly in the initial 4 h, which should derive from the quick release of the trapped electrons in the shallow traps (Trap I at 370 K and Trap II at 423 K). After 5 h decay, the ultraviolet emission signal becomes nearly undetectable, subsequent results are illustrated by the simulated curve (gray dotted line). As depicted by the time-dependent TL curves in the dark in Fig. 3b, when the phosphor decays naturally in the dark, the decrease of the TL intensity can be clearly observed only during the initial decay stage (1 min–3 h), which is consistent with the quick decrease of afterglow intensity in Fig. 3a. After 24 h natural decay in the darkness, even the shallow traps have not disappeared completely and the total TL integral intensity remains about 83% of the initial intensity, further demonstrating that the room-temperature thermal stimulation is not very sufficient to release the stored electrons in deep traps. Consequently, the results measured in the dark demonstrate that the ScBO_3_:Bi^3+^ phosphor is an excellent candidate for optical information storage, as it can effectively store X-ray excitation energy in the absence of external light stimulation at room temperature.

On the other hand, upon 100 lux white light illumination, the ultraviolet emission intensity exhibits a significant enhancement, as shown by the red solid line in Fig. 3a. In the initial stage over 0–4 h, the ultraviolet luminescence intensity undergoes a quick decay, followed by a slow decay over time. Even after 24 h of decay upon continuous white LED illumination, the ultraviolet luminescence intensity is still about two orders of magnitude higher than the background signal. The enhancement of ultraviolet light emission upon the illumination of polychromatic white light can be attributed to the continuous photo-release of the stored electrons in these deep traps (Trap III and IV). Figure 3c depicts the time-dependent TL curves upon 100 lux white LED illumination. The TL intensity at a certain decay instant decreases much faster compared with that in the dark. Even after 30 min of decay under the white light illumination, the TL intensity is already comparable to that after 24 h of natural decay in the dark, confirming the accelerated release of the trapped electrons under the continuous stimulation of white light. Moreover, unlike the TL curves in the dark, the TL intensities of Trap I and II show a significant decrease under white LED illumination. After 30 min of decay, the two shallow TL bands almost disappear. With the decay time increasing from 1 min to 24 h under white light illumination, the TL intensity shows a sharp declining trend and the TL peak persistently shifts to the higher temperature side. After 24 h decay under 100 lux white light illumination, the total TL intensity only remains around 15% of the initial intensity, which demonstrates the quick release of these deep-trapped electrons under the continuous photo-stimulation of polychromatic white LED. Furthermore, the ultraviolet emission spectra in the dark and under 100 lux white LED illumination were recorded at 1 h of decay after X-ray irradiation, as shown in Fig. 3d. The ultraviolet emission spectrum under 100 lux white LED illumination shares a similar profile with the photoluminescence emission spectrum (Fig. 1a), illustrating the white light stimulated ultraviolet light emission originates from the doped Bi^3+^ emitting centers. For comparison, we also recorded the emission spectrum of the ScBO_3_:Bi^3+^ phosphor in the darkness, as shown by the gray solid line in Fig. 3d. Unlike the intense ultraviolet luminescence under white light illumination, the ScBO_3_:Bi^3+^ phosphor in the dark exhibits an extremely weak afterglow emission.

To gain an in-depth understanding of the ultraviolet luminescence properties of the ScBO_3_:Bi^3+^ phosphor, low-temperature spectral experiments were conducted at 77 K. The ScBO_3_:Bi^3+^ phosphor was exposed to X-ray irradiation at room temperature and then immediately transferred to a liquid-nitrogen-filled cryostat, which can set the temperature at the range of 77–500 K. As shown in Fig. 3e, the ultraviolet PersL decay curves upon darkness and white light illumination (100 lux) at both 77 K and room temperature were recorded. When measured at 77 K in the dark, no ultraviolet luminescence signal can be observed, indicating that the stored electrons in energy traps cannot be released without thermal stimulation or light stimulation. However, ultraviolet light emission exhibits a significant enhancement when measured at 77 K under 100 lux white light illumination, which can be assigned to the continuous photo-release of the trapped electrons in the deep traps through the stimulation of the white light. Similarly, the emission intensity at room temperature under 100 lux white light illumination also shows a significant enhancement compared to that obtained in the dark, nearly matching with that observed at 77 K under 100 lux white light illumination. This further demonstrates that most trapped electrons are frozen at room temperature or 77 K, while external white light stimulation is efficient in releasing the stored electrons in the deep traps even at harshly low temperatures.

Figure 3f presents the repeated white light stimulated ultraviolet luminescence decay curve, with 3 min white light illumination and 2 min natural decay as a cycle. The sample was pre-irradiated by X-ray, followed by natural decay in the dark for 7 days at room temperature. After 7 days of natural decay, no ultraviolet emission signal can be detected. However, once turning on the white LED, the ultraviolet luminescence intensity increases by around 1000 times, indicating that the electrons in deep traps can be effectively released under white light stimulation. Moreover, the TL curve of the pre-irradiated ScBO_3_:Bi^3+^ phosphor after 7 days of natural decay in the dark at room temperature was also obtained, as shown in Fig. S7. After 7 days of decay, the two shallow traps disappeared and the total TL intensity remains at around 71% of the initial intensity, which can further prove the long-term storage performance of the ScBO_3_:Bi^3+^ phosphor in the dark. The above experimental results indicate that the photo-stimulation of white light can produce significant enhancement of ultraviolet luminescence in ScBO_3_:Bi^3+^ phosphor. However, there should be some energy loss in the photo-stimulated process and the optimal stimulating wavelength is yet unclear under the stimulation of polychromatic white light.

### Visible/NIR monochromatic light stimulated ultraviolet luminescence

**Fig. 4:**
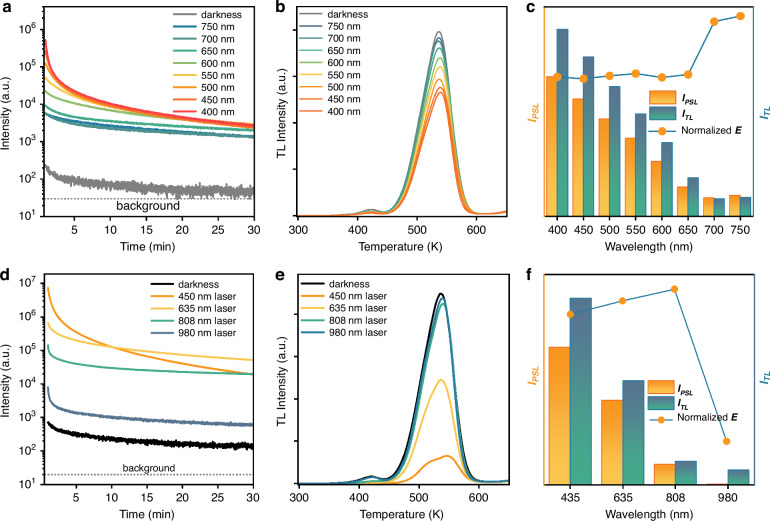
Visible/NIR monochromatic light stimulated ultraviolet luminescence. **a** Ultraviolet luminescence decay curves of the ScBO_3_:Bi^3+^ phosphor at room temperature under the photo-stimulation of different monochromatic lights over 400–750 nm. **b** TL spectra of the pre-irradiated ScBO_3_:Bi^3+^ phosphor after 30 min decay under continuous photo-stimulation of different monochromatic lights over 400–750 nm. The ScBO_3_:Bi^3+^ phosphor was first charged by X-ray for 25 min in the dark and immediately transferred to the spectrometer for testing, in which different stimulating wavelengths were applied. After 30 min decay, the TL curves were measured. **c** The integral of the enhanced ultraviolet luminescence intensity (*I*_*PSL*_) and the decreased TL intensity (*I*_*TL*_) along with the normalized effectiveness factor (*E*) of the pre-irradiated ScBO_3_:Bi^3+^ phosphor after photo-stimulation with different monochromatic lights. The effectiveness factor (*E*) is defined as *E* = *I*_*PSL*_/*I*_*TL*_. **d** Ultraviolet luminescence decay curves of the ScBO_3_:Bi^3+^ phosphor at room temperature under the photo-stimulation of different lasers (450, 635, 808, and 980 nm). **e** TL curves of the pre-irradiated ScBO_3_:Bi^3+^ phosphor after 20 min decay at room temperature with photo-stimulation of different lasers. **f** The integral of the enhanced ultraviolet luminescence intensity (*I*_*PSL*_) and the decreased TL intensity (*I*_*TL*_) along with the normalized effectiveness factor (*E*) of the pre-irradiated ScBO_3_:Bi^3+^ phosphor after photo-stimulation with different lasers. The effectiveness factor (*E*) is defined as *E* = *I*_*PSL*_/*I*_*TL*_

Considering the polychromatic compositions of white LED light (Fig. S8), it is necessary to evaluate the impact of different stimulating wavelengths on the ultraviolet luminescence. We measured the ultraviolet PersL decay curves of the ScBO_3_:Bi^3+^ phosphor under the stimulation of monochromatic light (Xenon lamp) over 400–750 nm with a step of 50 nm (the optical power of monochromatic light is the same.), as shown in Fig. 4a. In comparison to the natural decay curve in the dark, ultraviolet emission intensity realizes a significant enhancement under the photo-stimulation of these visible/NIR monochromatic lights. However, the effect of varied stimulating wavelengths on the luminescence process differs, shorter stimulating wavelengths lead to higher initial intensity but result in faster decay. As the stimulating wavelength increases, the remaining TL intensity shows an upward trend after 30 min of continuous stimulation, as displayed by the TL curves in Fig. 4b, illustrating that shorter stimulating wavelengths are more beneficial in liberating the stored electrons in these deep traps. However, the faster decay of the ultraviolet luminescence intensity under shorter-wavelength stimulation indicates that the released energy is not completely utilized for ultraviolet light emission, and energy loss should occur. Therefore, to investigate the effectiveness of photo-stimulation from different monochromatic lights, the effectiveness factor (*E*) is also introduced. Figure 4c exhibits the integral of the enhanced ultraviolet luminescence intensity (*I*_*PSL*_) and the decreased TL intensity (*I*_*TL*_) along with the normalized effectiveness factor (*E*) under varied stimulating wavelengths. As the stimulating wavelengths increase, both *I*_*TL*_ and *I*_*PSL*_ exhibit a downward trend, indicating that the shorter-wavelength light can release more stored electrons. At the same time, the normalized *E* shows a tiny fluctuation under the continuous photo-stimulation of monochromatic light with wavelengths ranging from 400 to 650 nm. However, it rises sharply when the monochromatic light wavelength reaches 700 and 750 nm, indicating that light with a longer wavelength (>700 nm) can yield a more effective photo-release of these deep-trapped electrons.

The ultraviolet luminescence decay curves and TL curves monitored at 299 nm under the continuous stimulation of visible/NIR laser diodes (100 mW) at room temperature are shown in Fig. 4d, e. The measured results coincide with the conclusion obtained under monochromatic light stimulation from the Xenon lamp. Shorter stimulating wavelengths result in higher initial intensity but a faster decay rate. The normalized effectiveness factor (*E*) is also utilized, as exhibited in Fig. 4f. It shows that with the stimulating wavelength increasing from 450 to 808 nm, both *I*_*TL*_ and *I*_*PSL*_ decrease, but the normalized *E* shows an upward trend, and the optimal stimulating wavelength is determined to be 808 nm. However, when the laser wavelength is set at 980 nm, the stimulation is less effective, indicating that the 980 nm laser cannot effectively liberate the electrons stored in the deep traps. These results further demonstrate that the optimal stimulating wavelength should well correspond to the depth of the traps. Moreover, the ultraviolet luminescence decay curves and TL curves under the stimulation of 808 nm laser (6 mW) and white LED illumination (1000 lux) were also measured (Fig. S9). It was found that the 808 nm laser with relatively low output power can still yield a significant enhancement of the luminescence intensity, and the ultraviolet luminescence can last for more than 24 h (Fig. S10). Meanwhile, the TL intensity only diminishes very slightly, which further demonstrates that the 808 nm NIR laser is very effective in releasing these deep-trapped electrons with minimum energy loss.

### Thermally stimulated ultraviolet luminescence in ScBO_3_:Bi^3+^ phosphor

**Fig. 5:**
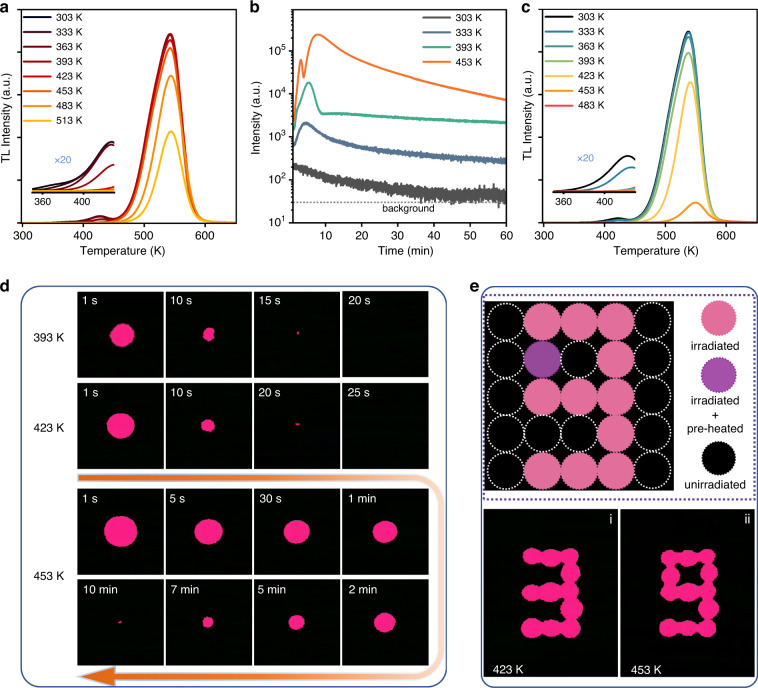
Thermally stimulated ultraviolet luminescence. **a** TL curve of samples undergoing thermal cleaning experiments for 60 s at various temperatures. **b** Ultraviolet PersL decay curves of the ScBO_3_:Bi^3+^ phosphor at different temperatures after irradiation by X-ray. **c** Temperature-dependent TL curves of the pre-irradiated ScBO_3_:Bi^3+^ phosphor after 60 min decay at a certain temperature. **d** Ultraviolet luminescence images of the ScBO_3_:Bi^3+^ phosphor disks recorded by the ultraviolet camera at different temperatures (393 K, 423 K, and 453 K). **e** Optical information storage induced by thermal stimulation. The top is a schematic illustration of the optical data storage application, the“pink” disc represents the irradiated samples (valid information); the“violet” disc represents the irradiated sample that was pre-heated at 423 K for 20 s (valid information); the “dark” disc represents the unirradiated samples (no information). The bottom is ultraviolet luminescence images of the ScBO_3_:Bi^3+^ phosphor disks heated at 423 K and 453 K, respectively

As an alternative to light stimulus, thermal stimulation at elevated temperatures can also trigger the release of these deep-trapped electrons effectively, resulting in enhanced ultraviolet light emission at high temperatures. To evaluate the release of the deep-trapped electrons in ScBO_3_:Bi^3+^ phosphor at different temperatures, the thermal cleaning experiment was carried out, as shown in Fig. 5a. The TL intensities of the Trap I (370 K) and II (423 K) decrease continuously at higher thermal cleaning temperature, and almost disappear at 363 K and 423 K, respectively, as shown in the inset of Fig. 5a. In contrast, the TL intensities of deep traps (Trap III and IV) remain nearly invariable until 423 K. As the temperature continues increasing, TL intensities decrease significantly accompanied by a gradual shift of the peak maximum to a higher temperature, indicating the effective release of the deep-trapped electrons at elevated temperatures (>423 K). These results illustrate that the ScBO_3_:Bi^3+^ phosphor can effectively store electrons at or above room temperature (<423 K), and only release them effectively at enough high temperature (>423 K), resulting in enhanced ultraviolet luminescence above a certain temperature.

Figures 5b and S11 show the ultraviolet PersL decay curves of the ScBO_3_:Bi^3+^ phosphor at different temperatures. The sample was pre-irradiated with X-ray for 25 min at room temperature and then transferred to a liquid-nitrogen-filled cryostat with a set temperature. Since the sample temperature requires time to increase from room temperature to the set temperature, there will be a rising process in the initial stage of the decay curve, which can be attributed to the accelerated release of the trapped electrons under thermal stimulation. Due to the unique trap distribution and various heat transfer rates, the decay curves of the ScBO_3_:Bi^3+^ phosphor exhibit different “rising” behaviors in the initial stage at different temperatures. At 333 K, the decay curve exhibits a single uptrend, which can be attributed to the release of the electrons in Trap I (370 K). When the set temperature continues to increase to 393 K, the decay curve shows two obvious rise processes with different rising rates, which should be assigned to the release of the electrons in Trap I (370 K) and Trap II (423 K), respectively. Furthermore, as the temperature rises higher (453 K), the decay curves show a complicated “rise-drop-rise” trend. The first “rise” stage can be attributed to the fast release of the electrons in the shallow traps (Trap I and II). Owing to the low density of the shallow traps, electrons are emptied quickly, while at this moment, the electrons in the deep traps cannot be effectively released, causing the “drop” process. Subsequently, the sample temperature continues to rise to effectively release these deep-trapped electrons, resulting in the second “rise” stage. After undergoing different rising behaviors in the initial stage, the sample temperature reaches the set temperature, meaning the beginning of the natural decay at the set temperature. It is noted that with the set temperature increasing, the “beginning intensity” becomes higher accompanied by a faster decay, indicating the faster release of the trapped electrons at higher temperatures. The temperature-dependent TL curves of the pre-irradiated ScBO_3_:Bi^3+^ phosphor after 60 min decay in Fig. 5c are in agreement with the results. After 60 min decay at 333 K, only the TL intensities of the low-temperature side (shallow traps) decrease, and the TL intensities of deep traps remain almost unchanged, illustrating that the thermal stimulation from 333 K can only accelerate the release of the trapped electrons from shallow traps. As the set temperature rises to 363 K and 393 K, the shallow traps disappear completely after 60 min decay and the TL intensities of the deep traps exhibit a small decrease. As the temperature continues to rise, the TL intensities of deep traps drop apparently and the peak maximum of the TL curve gradually shifts to a higher temperature. After decaying 60 min at 483 K, a majority of stored electrons were emptied. These results further demonstrate the excellent storage performance of the ScBO_3_:Bi^3+^ phosphors.

Furthermore, the thermally stimulated ultraviolet luminescence of the pre-irradiated ScBO_3_:Bi^3+^ phosphor disks was also visually recorded using an ultraviolet camera (Fig. S12). Given the deep-trapped electrons in energy traps and the sensitivity limitations of the camera, a temperature threshold is necessary to record the visualized ultraviolet luminescence. As shown in Fig. S12, the ultraviolet luminescence signal (pink area) starts to appear until 393 K, indicating the thermal stimulation at this temperature is enough to yield ultraviolet luminescence that can be captured by our camera. The higher the temperature, the bigger the pink area, illustrating the stronger ultraviolet PersL, which matches well with the results in Fig. 5b, c. Figure 5d presents the ultraviolet luminescence images of the ScBO_3_:Bi^3+^ phosphor disks at 393 K, 423 K, and 453 K for a long time (timing starts from the appearance of the pink area). At 393 K and 423 K, the pink area disappeared after decaying for 20 s and 25 s, respectively. In contrast, the pink area can still be observed after 10 min decay at 453 K, indicating the thermal stimulation at 453 K can effectively release the stored electrons in the deep traps and yield strong ultraviolet luminescence for a long time. By utilizing different ultraviolet PersL performance at varied temperatures, an optical information storage experiment was designed, as depicted in Fig. 5e. The top is a schematic illustration of the optical data storage application, the digital number “9” is written into a 5 × 5 ScBO_3_:Bi^3+^ phosphor disks array by X-ray irradiation, the“pink” disc represents the irradiated sample (valid information); the“violet” disc represents the irradiated sample that was pre-heated at 423 K for 20 s (valid information); the“dark” disc represents the unirradiated sample (no information). After the write-in process by X-ray, the wrong information “3” can be observed at 423 K due to the thermal stimulation is not enough to stimulate the “violet” disc to yield visual ultraviolet luminescence. However, at 453 K, the valid information “9” can be read out for a long time (more than 10 min, see Fig. 5d) owing to the sufficient thermal stimulation. The above results illustrate that the ScBO_3_:Bi^3+^ storage phosphor could be an excellent candidate for anti-counterfeiting applications in harsh environments.

### The nature of energy traps and ultraviolet luminescence mechanism

**Fig. 6:**
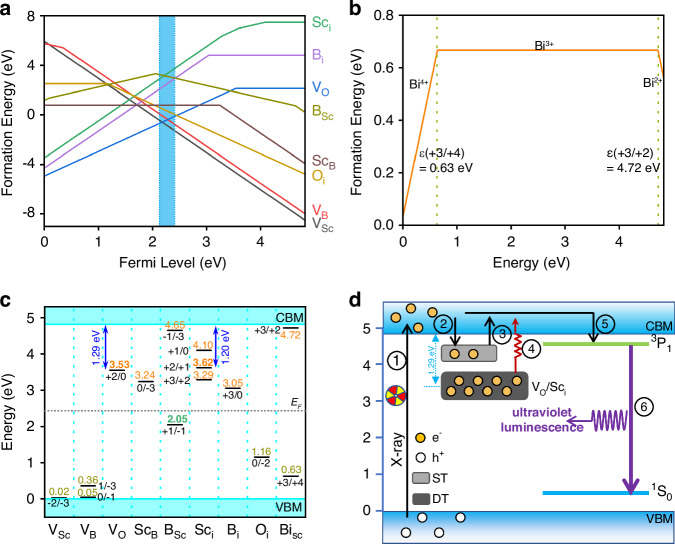
The nature of energy traps and ultraviolet luminescence mechanism. **a** The formation energies of intrinsic point defects with various charge states as a function of the Fermi level (*E*_*F*_) position within the band gap of ScBO_3_. **b** The formation energy of the Bi dopant at different charge states in the ScBO_3_ host. **c** The defect levels of Bi^3+^ dopant and the intrinsic defects in the ScBO_3_ host determined through the charge transition level calculations. **d** Ultraviolet luminescence mechanism in the ScBO_3_:Bi^3+^ phosphor

To shed more light on the ultraviolet luminescence mechanism, it is necessary to study the roles of specific types of defects in the trapping and de-trapping processes. By the DFT calculations, we have considered the influence of the possible intrinsic point defects, including vacancies (V_Sc_, V_B_, V_O_), antisites (Sc_B_, B_Sc_), and interstitials (Sc_i_, B_i_, and O_i_), along with impurity point defect, substitution (Bi_Sc_) in the ScBO_3_:Bi^3+^ phosphor. For the position of interstitials, we consider four different sites in the ScBO_3_ crystal structure (Fig. S13), and the results obtained for the most stable configuration were adopted. To assess the relative thermodynamic stabilities of various charge states of these defects, we evaluated the defect formation energies as a function of the Fermi level (*E*_*F*_) within the band gap, as shown in Fig. 6a. As the phosphors were synthesized in an air environment, their corresponding *E*_*F*_ positions were somewhat lower than the middle position of the band gap^53^, we concentrate on the results related to the E_F_ level to within 0.3 eV below the intermediate position, as seen by the blue shaded regions in Fig. 6a. For the ScBO_3_:Bi^3+^ phosphor, 20% excess of H_3_BO_3_ was added during the synthesis. Under this condition, the most probable intrinsic defect is V_Sc_^3−^, followed by V_B_^3−^, V_O_^2+^, O_i_^2−^, Sc_B_^0^, B_i_^3+^, B_Sc_^1−^, and Sc_i_^3+^. It is true that the lower the formation energies, the easier it is for defects to form, thereby the interstitial O_i_, the antisite Sc_B_, and the vacancies V_Sc_, V_B_, V_O_ are the main defects due to the relatively low formation energy but other intrinsic point defect are hardly formed because of the high formation energy (>2 eV). However, owing to the elastic collision of high-energy photons with the component atoms during X-ray irradiation, their occurrence probability would still be taken into consideration.

Besides, we have also calculated the effect of the introduction of Bi^3+^. The self-redox nature of Bi^3+^ allows it to function as both an emission center and a charge-carrier trapping center, meaning it may capture a hole to become a Bi^4+^ or an electron to become a Bi^2+^^54^. Figure 6b shows the defect formation energy of the substitution (Bi_Sc_) in the ScBO_3_:Bi^3+^ phosphor. It shows that when *E*_*F*_ is approximately in the middle region of the band gap, the 3+ charge state of Bi is the most stable state. The hole-trapping level ε(+3/+4), which represents the ground-state energy level of Bi^3+^^53^, is in a rather deep band gap with a depth of 0.63 eV above VBM, whereas the electron-trapping levels ε(+3/+2) that represent the ground-state energy level of Bi^2+^ inside the band gap is considerably shallower with the depth of 0.10 eV below CBM. Thus, Bi^3+^ can function as a shallow electron-trapping center in addition to a somewhat deep hole-trapping center in the ScBO_3_:Bi^3+^ phosphor.

Moreover, the thermodynamic charge transition levels for the aforementioned intrinsic point defects, along with the defect levels of Bi^3+^, were calculated, and the results are shown in Fig. 6c. The transition levels for a specific defect can be thought of as its energy level at various charge states, and their positions relative to the host VBM/CBM remain unaffected by the atomic chemical potentials associated with the defect. It shows that the antisite defect B_Sc_ ε(−1/−3) and Bi_Sc_ ε(+3/+2) transition levels may result in the creation of electron-trapping level in ScBO_3_, with the trap depths being about 0.17 and 0.10 eV below the CBM, respectively, which are so shallow that they will fully release at room temperature and cannot contribute to the ultraviolet PersL. While the transition level ε(+1/0) of interstitial defect Sc_i_ leads to the creation of an electron-trapping level with a depth of 0.72 eV, which will be responsible for the room-temperature thermally stimulated PersL. Also, the incorporation of interstitial defect Sc_i_ produces transition level ε(+2/+1) at a depth of 1.20 eV and the vacancy defect V_O_ introduces transition level ε(+2/0) with a trap depth of 1.29 eV below the CBM, the two defect levels are both too deep to be stimulated by room temperature thermal energy. However, white light illumination or monochromatic light illumination with an appropriate wavelength or thermal stimulation at elevated temperatures can liberate the electrons trapped in these deep traps, giving rise to enhanced ultraviolet luminescence. Moreover, the antisite defect Sc_B_ ε(0/-3) transition level, interstitial defect Sc_i_ ε(+3/+2) transition level, and interstitial defect B_i_ ε(+3/0) transition level can also provide charge transition energy levels for deeper electron-trapping, with related trap depth of 1.58 eV, 1.53 eV and 1.77 eV below the CBM, which are too deep to contribute to the PersL at room temperature. Actually, TL measurements of X-ray pre-irradiated ScBO_3_:Bi^3+^ phosphor display four glow peaks, and the dominant TL bands are located at about 522 K (Trap III) and 547 K (Trap IV). Using the improved formula by Liu group^55^, *E*_*depth*_ = (−0.94ln*β* + 30.09)*kT*_*m*_, where *E*_*depth*_ denotes the trap depth, *β* presents the heating rate, *k* is the Boltzmann constant and *T*_*m*_ is the temperature at the peak (K), the estimated trap depths (Trap III and IV) are 1.29 and 1.35 eV, respectively. Comparing the experimental data with the DFT calculations (Fig. 6c), one can see that, the deep traps (Trap III and Trap IV) probably arise from Sc_i_ ε(+2/+1) transition level (1.20 eV) and V_O_ ε(+2/0) transition level (1.29 eV), respectively. Besides, we have tested the EPR spectra of the raw sample and the sample after X-ray irradiation (Fig. S14). Following X-ray exposure for 25 min, a distinct EPR signal at *g* = 2.003 can be observed, indicating the formation of V_O_, which serves as electron traps in the ultraviolet persistent luminescence process, further demonstrating the reliability of the DFT calculations.

Based on the aforementioned results and discussions, we have proposed a possible mechanism to interpret the enhanced ultraviolet luminescence of the ScBO_3_:Bi^3+^ phosphor in bright environments, as shown in Fig. 6d. To streamline the discussion, we assign the deep traps and the shallow traps as DT and ST, respectively. When excited by high-energy X-ray, electrons in the valence band (VB) will be promoted to the CB, leading to the creation of holes in the VB (process 1)^56^. A portion of electrons can be effectively captured by energy traps, which are primarily lattice intrinsic defects Sc_i_ ε(+2/+1) and V_O_ ε(+2/0) transition levels that are located below the bottom of CB, and the holes in the VB will be captured by B_Sc_ ε(+1/−1) transition level (2.05 eV, process 2). When the X-ray excitation is switched off, the electrons trapped within the ST and DT can be released into the CB by room-temperature thermal stimulation (process 3). The electrons in the CB must relax to the bottom of the CB, and then recombine with the Bi^3+^ emitter (process 5), leading to the ultraviolet luminescence (process 6). However, for the ScBO_3_:Bi^3+^ phosphor, only a few electrons are captured in the ST and the depth of the DT is large enough, the thermal stimulation at room temperature cannot effectively liberate these deep-trapped electrons (Fig. 3b), thereby the ultraviolet PersL in the dark at room temperature is extremely weak. On the other hand, with the aid of visible/NIR photo-stimulation or high-temperature thermal stimulation, the trapped electrons in the DT could be effectively released into the CB (process 4), resulting in considerable enhancement of ultraviolet light emission by employing interplay between defect levels and external stimulus. It is concluded that the effectiveness of stimulating wavelength should be related to the depth of the traps. Upon the stimulation of the polychromatic white LED light, the trapped electrons can be pumped into the CB, and the stored energy is released, but the effectiveness is far below monochromatic light (Fig. S9). The 980 nm light can release the stored energy in the shallow traps (Trap I and II), and realize a small enhancement due to the low density of these traps, while the 808 nm laser can effectively liberate the stored electrons in the deep traps (Trap III and IV) to the bottom of the CB, resulting in minimal energy loss and an enhanced ultraviolet light emission. However, for the 635 nm and 450 nm visible light, the energy is high enough to release the electrons in the deep traps and will promote more electrons to reach the inside of the CB, and then the electrons will relax to the CB bottom, resulting in the loss of a significant amount of stored energy. In addition, the measured results of the YBO_3_:Bi^3+^ phosphors also demonstrate that the interplay between the optimal stimulating wavelength (980 nm) and defects produces a more effective enhancement of ultraviolet light emission (Fig. S15).

### Optical information storage application of the ScBO_3_:Bi^3+^ phosphor

**Fig. 7:**
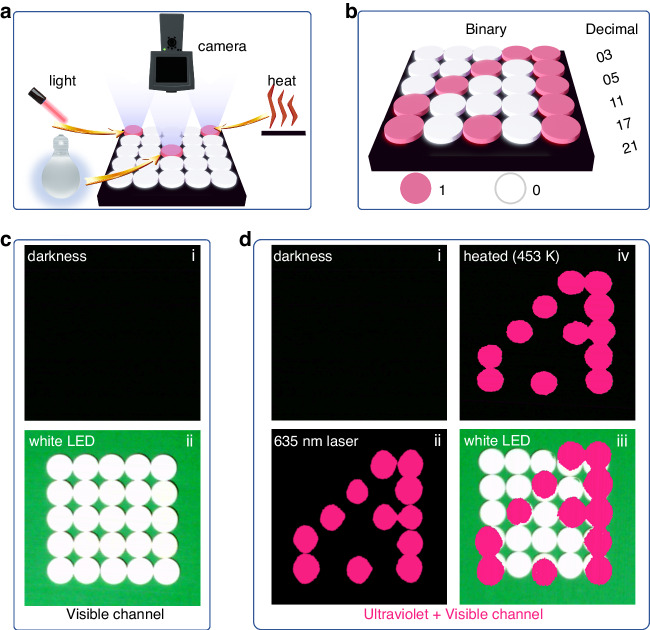
Demonstration experiment for advanced optical information storage application. **a** A schematic illustration of optical information read-out experiment. **b** The designed binary information and corresponding decimal information. **c** Photos of the phosphor disks array by naked eye upon darkness (inset i) and white LED illumination (inset ii). **d** Photographs of the phosphor disks array read out by the ultraviolet camera at room temperature upon darkness (inset i), darkness +635 nm laser (10 mW) (inset ii), indoor white LED light (300 lux) (inset iii) and 453 K heating (inset iv)

Taking into account the lack of deep ultraviolet background signal in indoor ambient lighting conditions and the exceptional photo-stimulated and thermally stimulated ultraviolet luminescence feature of the ScBO_3_:Bi^3+^ phosphor, the optical information storage applications can be expected. Figure 7a shows a schematic illustration of optical data write-in and read-out experiment by using the ScBO_3_:Bi^3+^ phosphor disks. To validate the feasibility of the optical write-in and read-out scheme, as well as to assess the storage performance of the ScBO_3_:Bi^3+^ phosphor, we showcase the binary information encoding using X-ray and decoding using light stimulus (white LED or 635 nm red laser diode) and heat stimulus. Five decimal values are converted to binary, with one decimal number represented by each line and the binary information is encoded into a 5×5 ScBO_3_:Bi^3+^ phosphor disks array after X-ray irradiation, as depicted in Fig. 7b. After writing-in, no signal can be detected by the naked eye in the darkness or under white light illumination (Fig. 7c). However, using the ultraviolet camera, the read-out information is well reproduced with the photo-stimulation of polychromatic white light (300 lux) or monochromatic 635 nm red light (10 mW) in bright and dark environments (Fig. 7d (ii) and (iii)), or under the thermal stimulation at 453 K (Fig. 7d (iv)), which enables the controllable optical information read-out under specific external conditions. In addition, an optical information encryption application has also been demonstrated (Fig. S16). Two groups of 2 × 2 unit arrays were designed into different information, and 16 results of the optical information read-out based on the array can be obtained. The only way to get valid information is to have the only correct way to decrypt it, which greatly enhances the security of information. These results indicate the feasibility of our scheme and showcase the excellent optical information storage performance of the ScBO_3_:Bi^3+^ phosphor. Besides, it should be noted that the as-synthesized ScBO_3_:Bi^3+^ phosphor exhibits excellent moisture resistance, luminescence stability, write-erase stability, and reproducibility (Figs. S17–20), endowing it the potential for practical and robust optical storage applications.

## Discussion

In summary, we report a novel deep-trap ultraviolet persistent phosphor, ScBO_3_:Bi^3+^, whose emission peak is located at 299 nm with a record FWHM of only 0.21 eV. The effects of the spectral distribution of ambient light and ambient temperature on the ultraviolet light emission process have been studied in detail. On this basis, intense and long-lasting ultraviolet luminescence is realized by subjecting the phosphor to continuous photo-stimulation of white/NIR light illumination, as well as high-temperature thermal stimulation by employing the interplay between defect levels and external physical stimulus (light or heat), even though the ultraviolet light emission in the dark at room temperature is extremely weak. Through comprehensive experimental and theoretical analyses, we have identified the involvement of both O vacancy and Sc interstitial for the prolonged and enhanced ultraviolet luminescence in bright environments. In addition, we demonstrate that covert luminescence information can only be read out under specific light or thermal stimulation, showcasing the potential for controllable optical data storage applications. Overall, this study not only inspires the discovery of more glow-in-the-daylight materials featuring deep traps and deep ultraviolet luminescence but also contributes new insights into the underlying luminescence mechanisms of persistent phosphors in bright environments.

## Materials and methods

Sc_2_O_3_ (99.99%, Aladdin), H_3_BO_3_ (99.99%, Aladdin), and Bi_2_O_3_ (99.99%, Aladdin) were used as the starting chemicals. All the powders were used without further treatment. ScBO_3_:*x*%Bi^3+^ (0 < *x* ≤ 1) storage phosphors were synthesized *via* a high-temperature solid-state reaction method. Accurate weighing of stoichiometric amounts of raw materials was carried out, with an addition of 20% excess of H_3_BO_3_ was added to offset evaporation loss during the high-temperature sintering. The raw materials were thoroughly ground in an agate mortar, followed by a pre-firing at 873 K for 2 h at a heating rate of 10 K/min in air. After that, the pre-fired powders underwent additional grinding and were then pressed into disks with a diameter of ~10 mm at a pressure of 7 MPa. Finally, the resulting disks were sintered at 1473 K for 5 h at a heating rate of 10 K/min in an air environment and cooled naturally to room temperature. The structure, morphology, persistent luminescence, PSL, and thermoluminescence properties of the phosphors were measured and analyzed in detail. The DFT was used to investigate the nature of defects by using the Vienna Ab initio simulation package (VASP). Characterization and computational details are shown in Supporting Information.

## Supplementary information

Supplemental Material
