# Supplementary material for: Deep-trap ultraviolet persistent phosphor for advanced optical storage application in bright environments
Source: Light Sci Appl. 2024 Sep 14;13:253. doi: 10.1038/s41377-024-01533-y (PMC11401881; doi:10.1038/s41377-024-01533-y)
Supplement: Supplementary file 1 — Supplemental Material [file 41377_2024_1533_MOESM1_ESM.pdf]

Supporting Information

Deep-trap ultraviolet persistent phosphor for advanced optical storage application in bright environments

Xulong Lv[1], Yanjie Liang[1*], Yi Zhang[1], Dongxun Chen[1], Xihui Shan[1], Xiao-Jun Wang[2*]

[1]Key Laboratory for Liquid-Solid Structure Evolution and Processing of Materials, Shandong University, Jinan 250061, China

[2]Department of Physics, Georgia Southern University, Statesboro, GA 30460, USA

Correspondence: Yanjie Liang (yanjie.liang@sdu.edu.cn)

Xiao-Jun Wang (xwang@georgiasouthern.edu)

Materials and methods

Characterization

The crystal structure of the as-synthesized phosphors was characterized *via* powder X-ray diffraction (XRD, DMAX-2500PC, Rigaku) with Cu Kα irradiation (λ = 1.5418 Å) at a scanning speed of 10° min^{-1}. The morphology and elemental distribution of the samples were recorded by a JSM-7800F field-emission scanning electron microscope (SEM). The photoluminescence (PL), persistent luminescence (PersL), and photostimulated luminescence (PSL) properties were measured using an FLS1000 spectrofluorometer (Edinburgh Instruments) equipped with a photomultiplier tube detector (PMT, 200-900 nm) and a 400 W Xe lamp as the excitation source. The X-ray source (MOXTEK MagPro) was used as an excitation source. The low-temperature and high-temperature experiments were realized with an OptistatDN cryostat (Oxford Instruments) equipped with a MercuryiTC temperature-controlled system. Thermoluminescence (TL) spectra were conducted by using an SL18 thermoluminescence setup (Guangzhou Rongfan Science and Technology Co., Ltd; heating rate, 4 K s^{-1}). The ultraviolet afterglow images were taken by the ultraviolet camera (Ofil Scalar), which are overlay images after the addition of a ultraviolet image onto a visible image. The PSL measurements and imaging experiments are carried out in an indoor-lighting environment (under the illumination of a Bull white LED lamp). The electron paramagnetic resonance (EPR) spectra were obtained using a Bruker A300 spectrometer.

Computational details

Parameter settings

The initial atomic positions and symmetry information of the $ScBO_3$ crystal structure were taken from the Inorganic Crystal Structure Database and the periodic 2 × 2 × 1 supercell containing 120 atoms (24 Sc atoms, 24 B atoms and 72 O atoms) was used to simulate. Using the Vienna Ab initio simulation package (VASP),[1] theoretical simulations were carried out using the density

functional theory (DFT), and the generalized gradient approximation (GGA)-Perdew-Burke-Ernzerhof (PBE) exchange-correlation functional was adopted. The Sc ($3s^2 3p^6 4s^1 3d^2$), B ($2s^2 2p^1$), O ($2s^2 2p^4$) and Bi ($5d^{10} 6s^2 6p^3$) were treated as valence electrons, and their interactions with the cores were described by the projector augmented wave (PAW) method. The energy change and the Hellmann-Feynman forces on atoms were set to 10^{-5} eV and 0.01 eV Å^{-1}, respectively. The plane-wave cut-off energy was set at 550 eV. For k-point integration within the first Brillouin zone, a 3 × 3 × 3 Monkhorst–Pack grid was selected.

Formation energy and charge transition levels

The value of defect formation energy reflects the ease of defect formation and the stability of the defect system. The formation energy of a defect in charge state q is defined by:

$$\Delta H_f = E_{tot}[D^q] - E_{tot}[host] - \sum_i n_i \mu_i + q(E_F + E_{VBM}) \quad (1)$$

where $E_{tot}[D^q]$ and $E_{tot}[host]$ are the total energies of the supercell with or without defect D with charge q; n_i means the change in number for atom i due to defect D, which is added to (n_i >0)or removed from (n_i <0) the perfect supercell; μ_i is the chemical potential for atom i; E_F is the electron Fermi energy; E_{VBM} is the energy state of the valence band maximum.

The atomic chemical potentials in eq (1) are correlated in the thermodynamic equilibrium:

$$\mu_{Sc} + \mu_B + 3\mu_O = \mu_{ScBO_3} \quad (2)$$

where μ_{ScBO_3} is the total energy of one formula unit of $ScBO_3$, and μ_{Sc}, μ_B, μ_O are the chemical potentials of Sc, B and O components, respectively. As the phosphors were synthesized in air, and 20% excess B was added during the synthesis, the atomic potentials were determined by the following expressions:

$$\mu_O = 1/2\mu_{O_2} + \Delta\mu_O \quad (3)$$

$$\mu_B = 1/2(\mu_{B_2O_3} - 3\mu_O) \quad (4)$$

$$\mu_{Sc} = \mu_{ScBO_3} - \mu_B - 3\mu_O \quad (5)$$

The thermodynamic charge-transition levels within the band gap correspond to the Fermi level position at which a transition occurs from one charge state (q) to another (q′). The level position ε(q/q') regarding the host VBM can be deduced from eq (1) as:

$$\varepsilon(q/q') = \frac{\Delta E_f(D^{q'}; E_F = 0) - \Delta E_f(D^q; E_F = 0)}{q - q'} \quad (6)$$

where ΔE_f (q or q′; E_F = 0) is the formation energy of the defect in charge state q or q′ when the Fermi level is set at 0 eV.

1. Kresse, G. & Furthmüller, J. Efficient iterative schemes for ab initio total-energy calculations using a plane-wave basis set. *Phys. Rev. B* **54**, 11169 (1996).

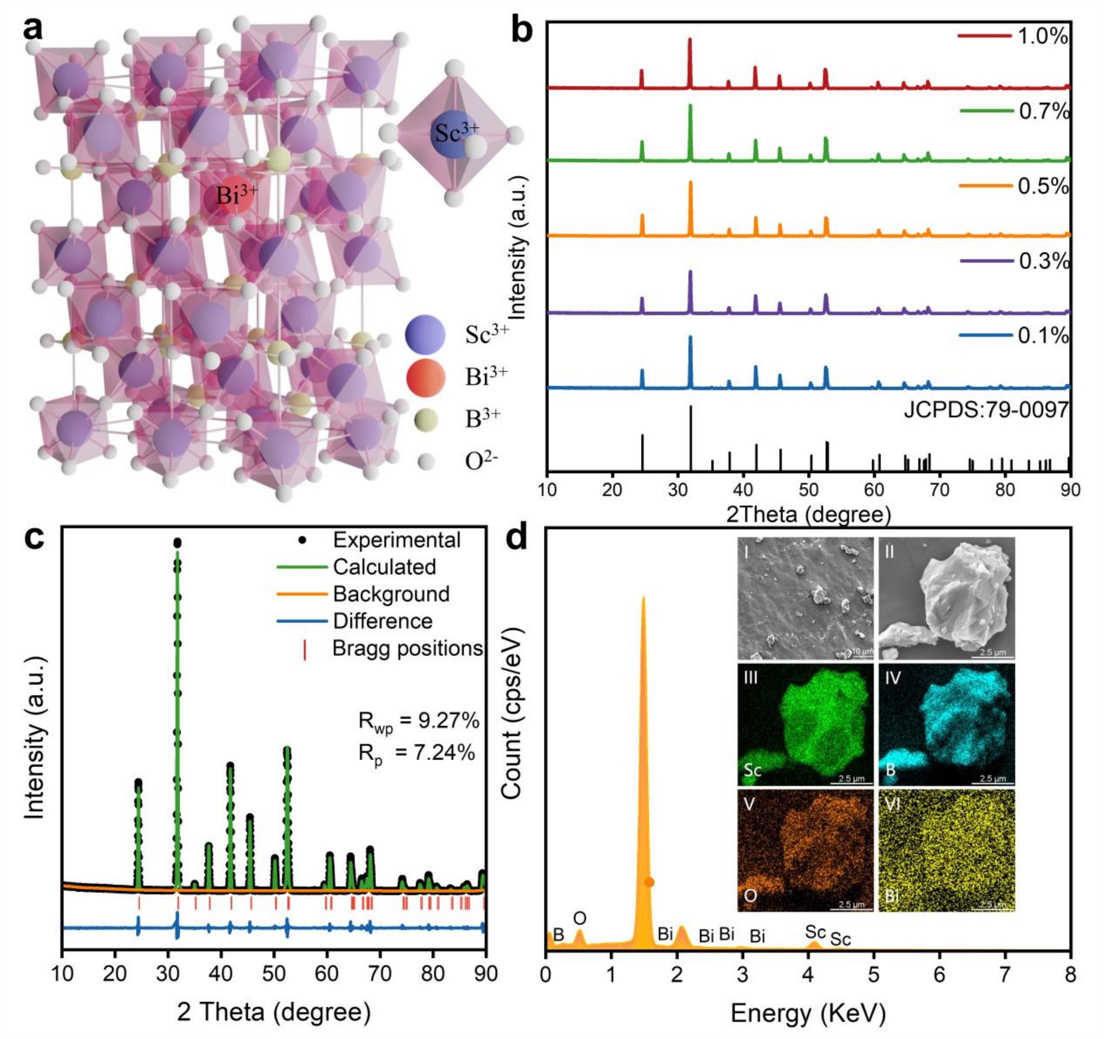

Fig. S1 Crystal structure and morphology of the $ScBO_3:Bi^{3+}$ phosphor. **a** A schematic diagram of the crystal structure of the $ScBO_3$ host. **b** XRD patterns of $ScBO_3$:x%Bi^{3+} phosphors ($0 < x \leq 1$). **c** The Rietveld refinement of the $ScBO_3:Bi^{3+}$ phosphor. **d** EDS spectrum of the $ScBO_3:Bi^{3+}$ phosphor. The insets show the SEM images and corresponding EDS mapping images of the $ScBO_3:Bi^{3+}$ phosphor.

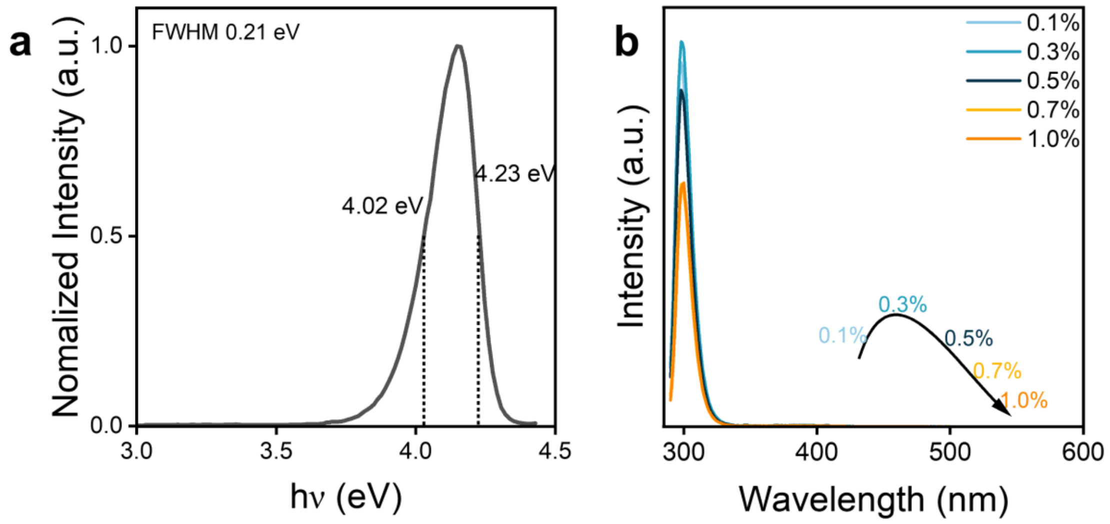

Fig. S2 **a** The emission spectrum of the $ScBO_3:Bi^{3+}$ phosphor with *hν (eV)* as a horizontal coordinate. **b** Photoluminescence emission spectra of the $ScBO_3:x\%Bi^{3+}$ ($0 < x \leqslant 1$) phosphors at room temperature. The emission spectra were obtained under the excitation of 280 nm UV light.

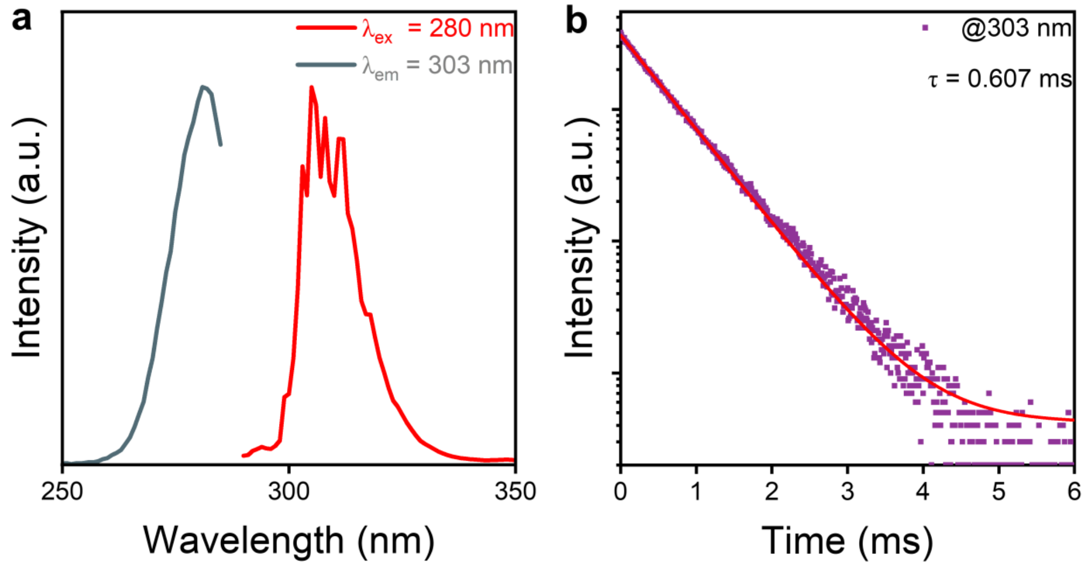

Fig. S3 a The normalized emission and excitation spectra of the $ScBO_3:Bi^{3+}$ phosphor at 77 K and room temperature. b The measured luminescence decay curve of the $ScBO_3:Bi^{3+}$ phosphor at 77 K.

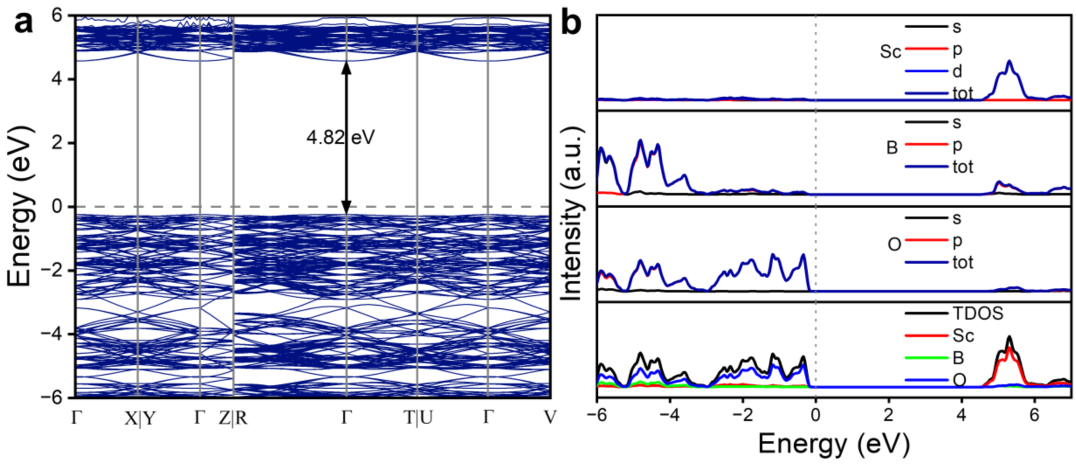

Fig. S4 a, b The calculated band structure and total DOS and partial DOS of the $ScBO_3$ host.

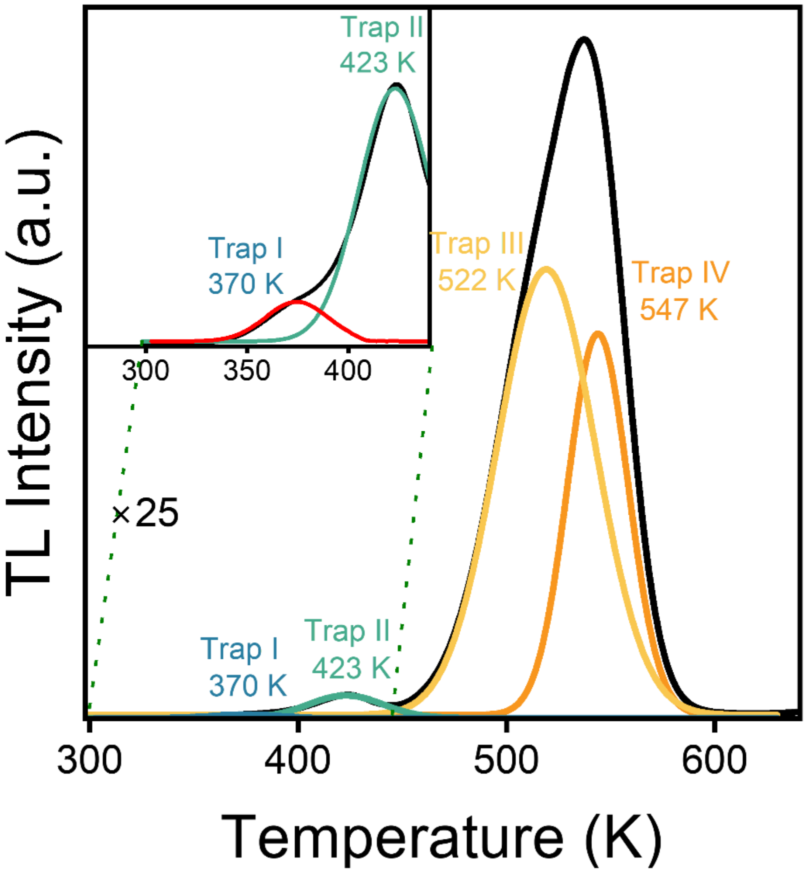

Fig. S5 The Gaussian fitting of the thermoluminescence spectrum of the $ScBO_3:Bi^{3+}$ phosphor and the value of the peaks are shown in the figure. The sample was pre-irradiated with an X-ray for 25 min.

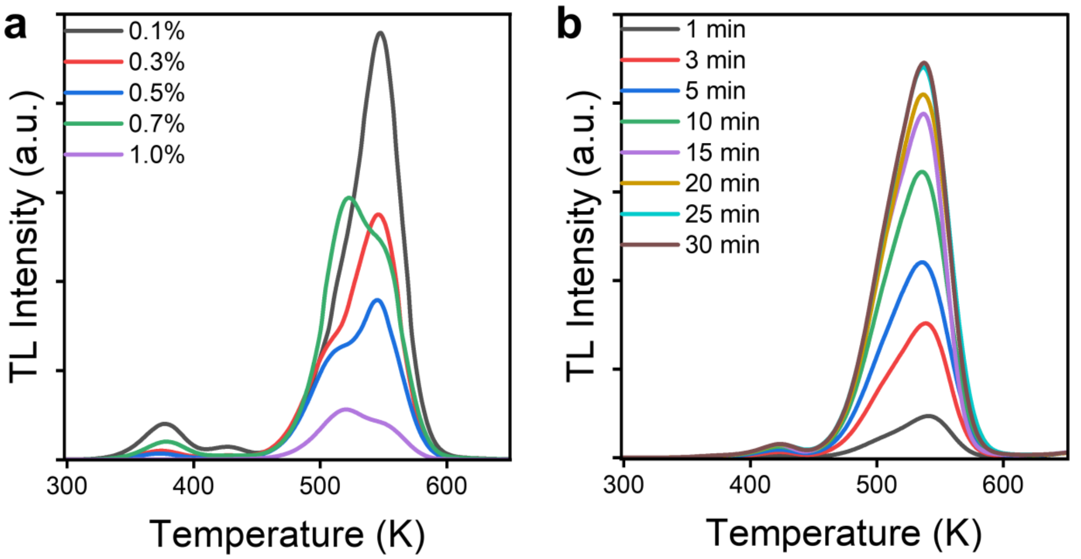

Fig. S6 a, b TL curves of the $ScBO_3:Bi^{3+}$ phosphors with different Bi^{3+} doping concentrations and different excitation durations. The TL curves were acquired at 60 s decay after ceasing X-ray irradiation.

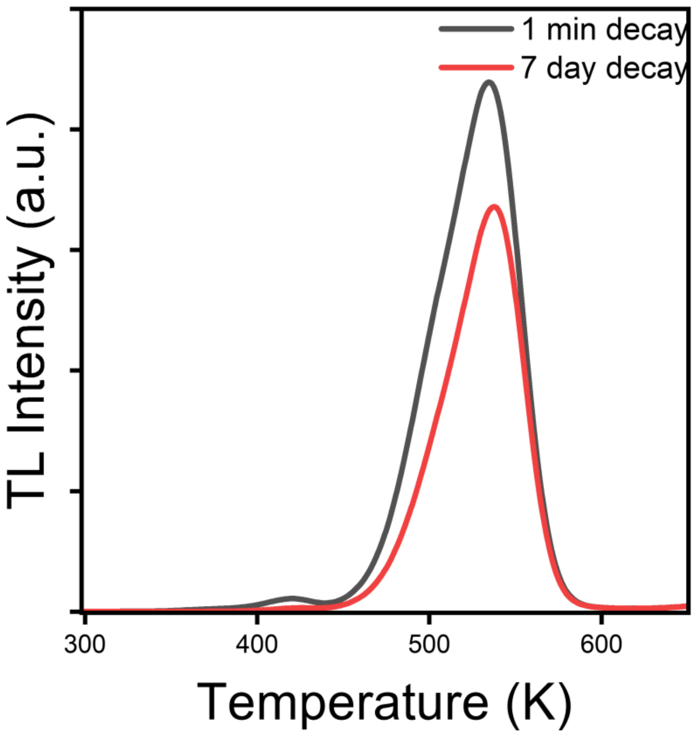

Fig. S7 TL curve of the pre-irradiated $ScBO_3:Bi^{3+}$ phosphor after 1min and 7 days of natural decay.

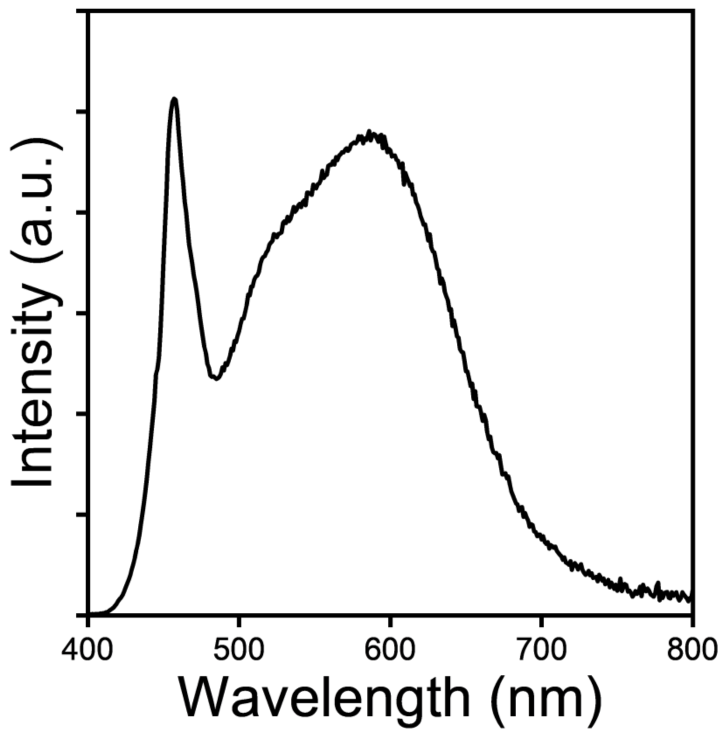

Fig. S8 Emission spectrum of the used white LED light source.

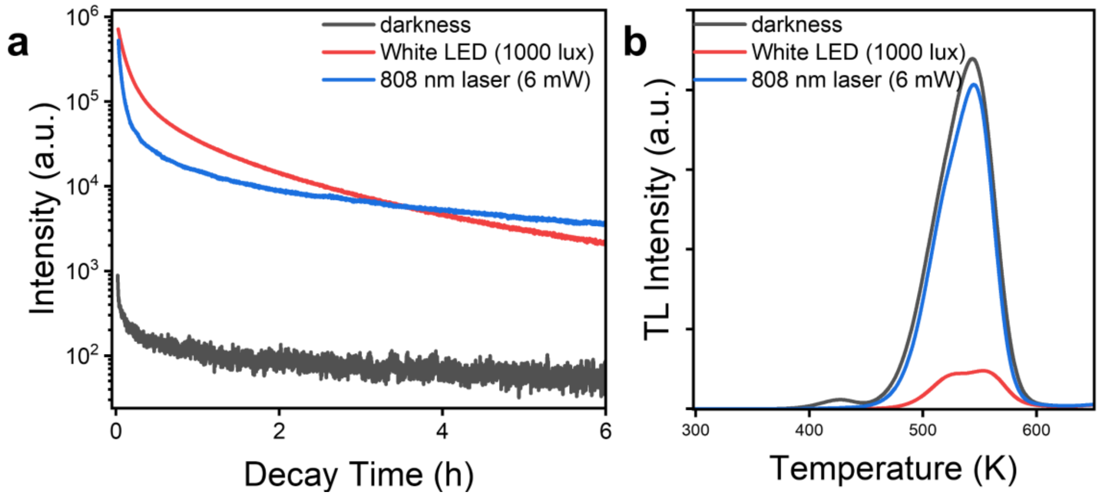

Fig. S9 **a** Ultraviolet luminescence decay curves of the $ScBO_3$:Bi^{3+} phosphor monitored at 299 nm after irradiation by X-ray for 25 min. The decay curves were measured under the stimulation of the 6 mW 808 nm laser and 1000 lux white LED illumination, respectively. **b** TL curves of the pre-irradiated $ScBO_3$:Bi^{3+} phosphor after 6 h decay under different ambient conditions (darkness, 1000 lux white LED, 6 mW 808 nm laser).

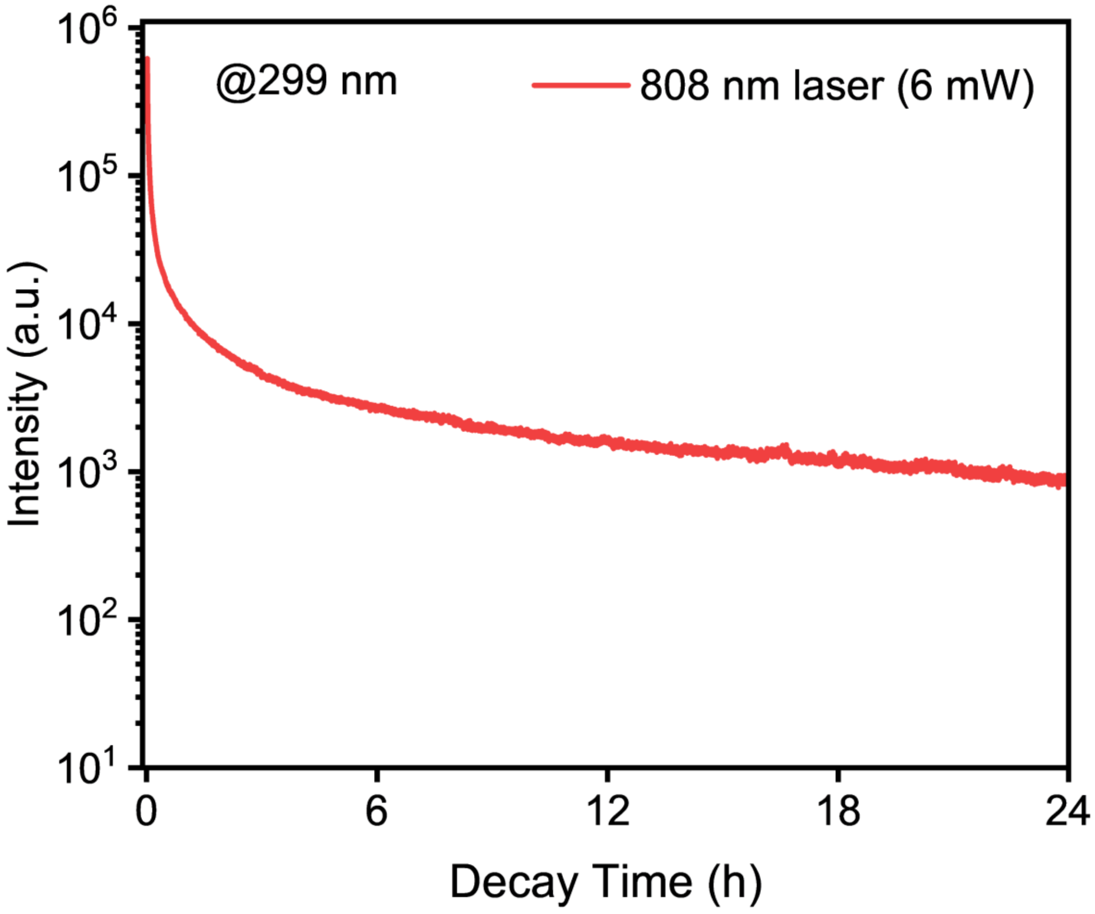

Fig. S10 Ultraviolet luminescence decay curve of the $ScBO_3:Bi^{3+}$ phosphor monitored at 299 nm under the stimulation of the 808 nm laser (6 mW) after irradiation by X-ray for 25 min.

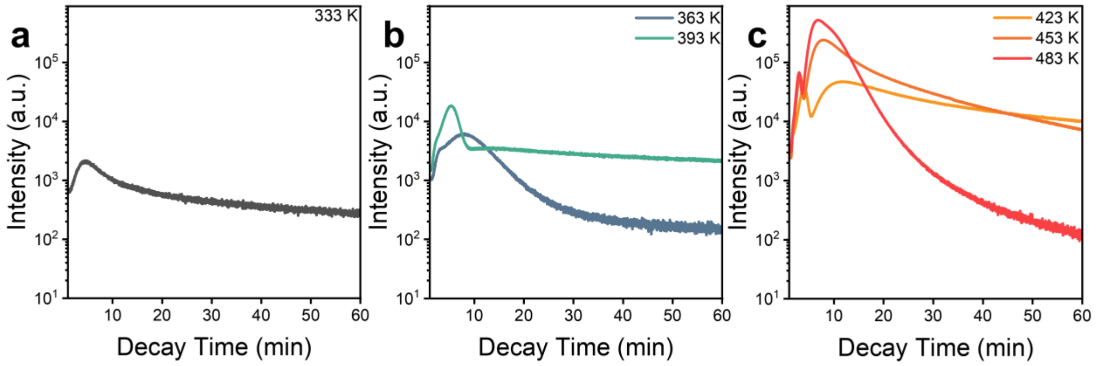

Fig. S11 a-c Ultraviolet luminescence decay curves of the $ScBO_3:Bi^{3+}$ phosphor at different temperatures after irradiated by X-ray for 25 min

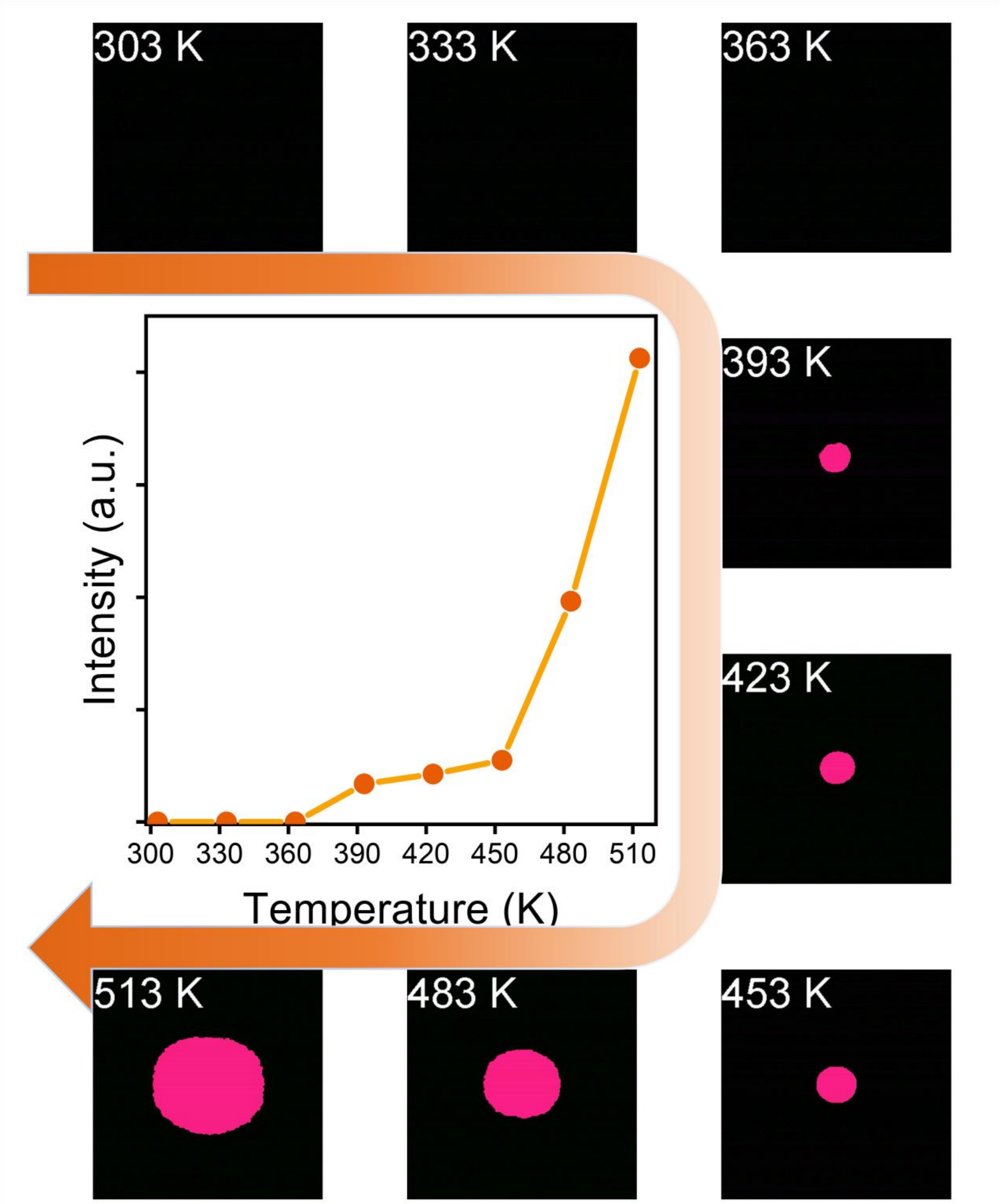

Fig. S12 Ultraviolet luminescence images of the $ScBO_3:Bi^{3+}$ phosphor discs at different temperatures.

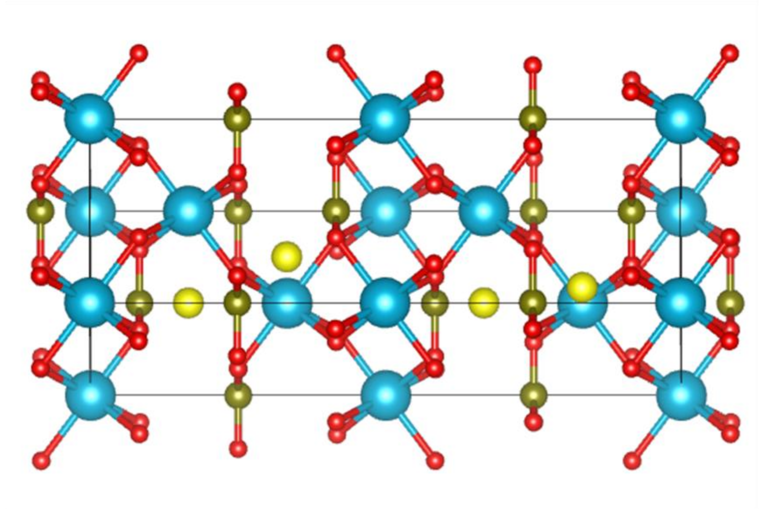

Fig. S13 The considered four different interstitial sites in the $ScBO_3$ crystal structure.

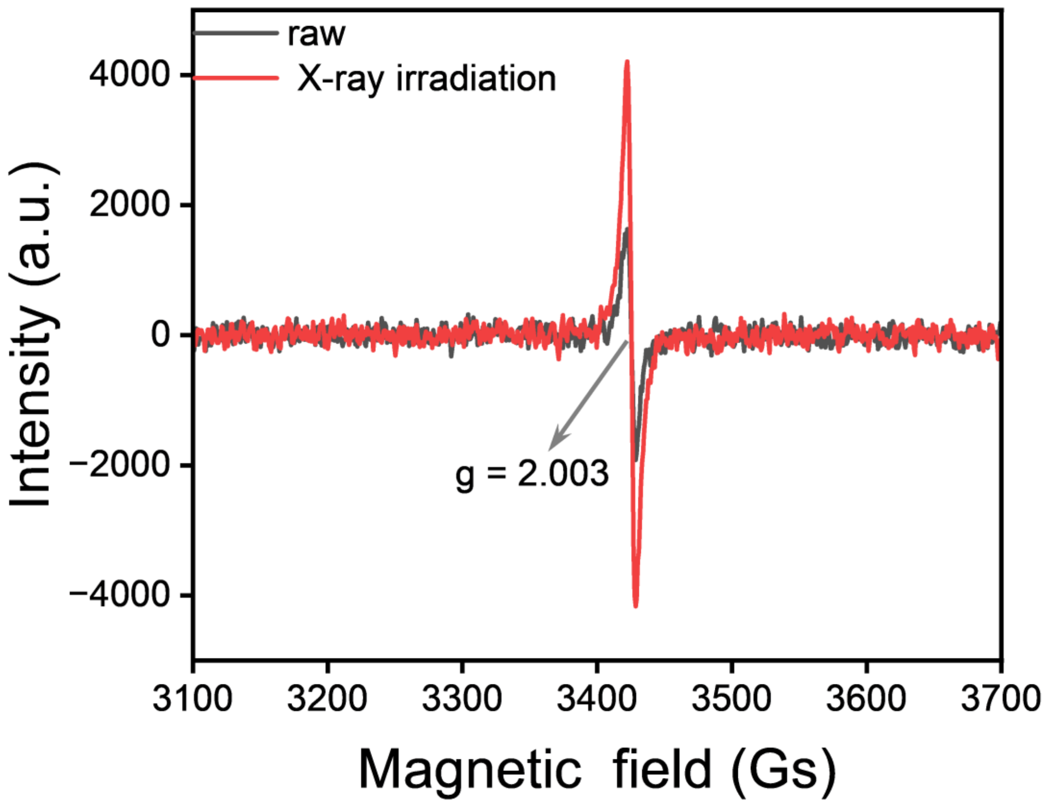

Fig. S14 EPR spectra of the unirradiated phosphor and the sample after X-ray irradiation.

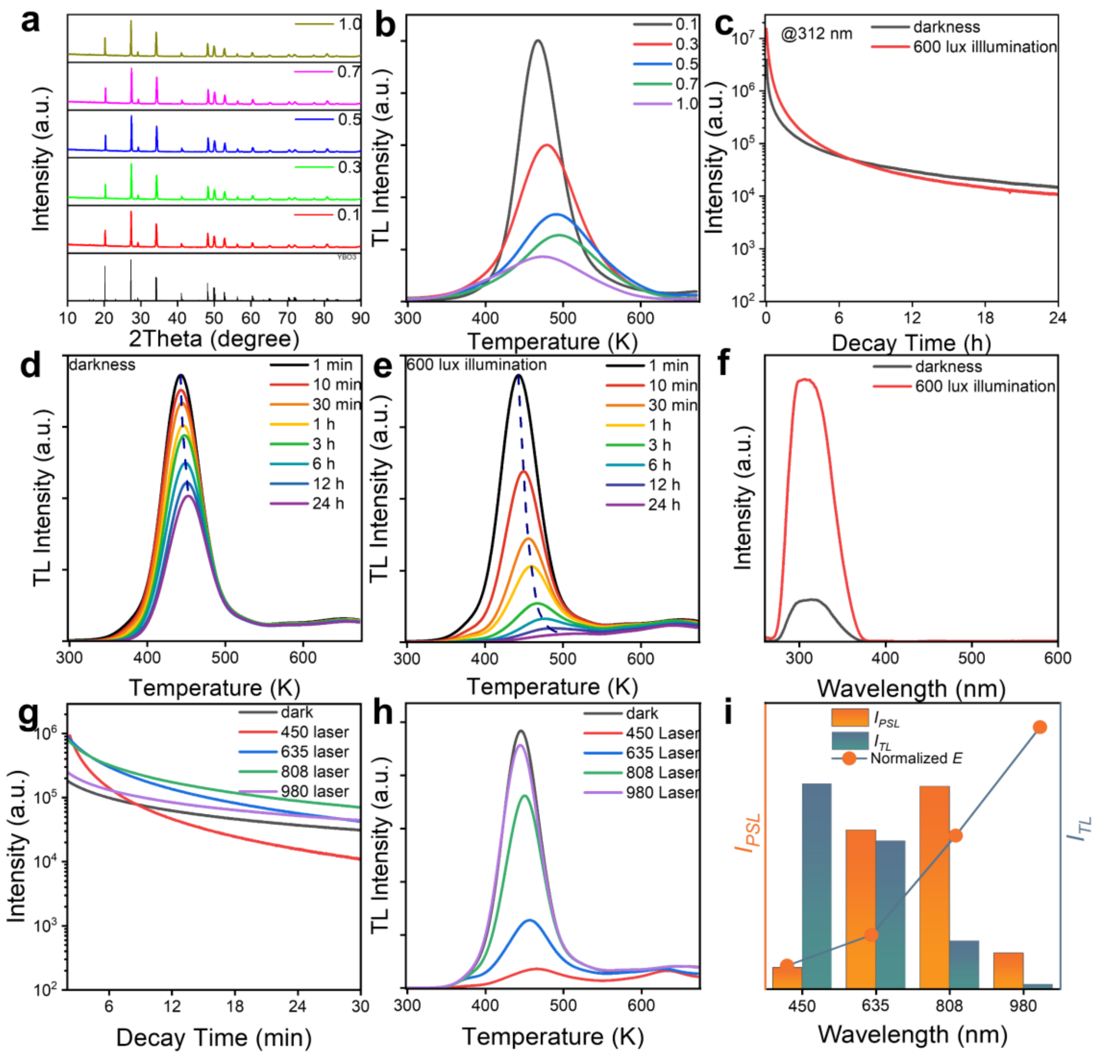

Fig. S15 a XRD patterns of YBO_3:x%Bi^{3+} phosphors ($0 < x \leqslant 1$). **b** TL curves of the YBO_3:x%Bi^{3+} phosphors ($0 < x \leqslant 1$). **c** PersL decay curves of the YBO_3:Bi^{3+} phosphor upon darkness and 600 lux illumination. **d, e** Time-dependent TL curves of the YBO_3:Bi^{3+} phosphor upon darkness and 600 lux white LED illumination. TL curves were acquired by monitoring at 312 nm over the range of 298 – 650 K. **f** The emission spectra acquired at 10 min decay upon darkness and 600 lux white LED illumination. **g** Decay curves of the YBO_3:Bi^{3+} phosphor at room temperature under photo-stimulation of different laser after irradiated by X-ray. **h** TL curves of the YBO_3:Bi^{3+} phosphor after 30 min decay in darkness with photo-stimulation of different lasers. **i** The integral of the enhanced luminescence intensity (I_{PSL}) and the decreased TL intensity (I_{TL}) along with the effectiveness factor (E) of the pre-irradiated YBO_3:Bi^{3+} phosphor after photo-stimulation of different lasers.

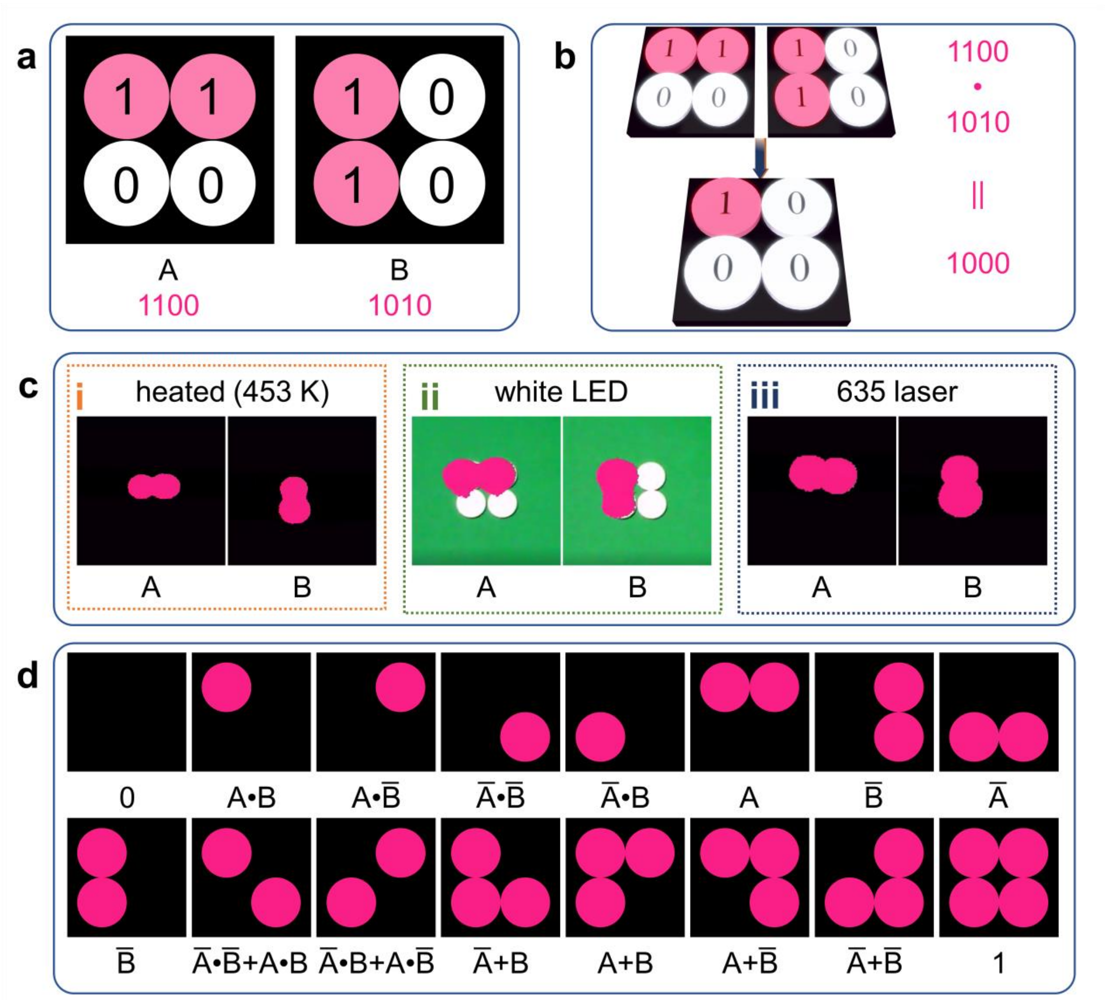

Fig. S16 The schematic of the optical information encryption. **a** The schematic of the input A (1100) and input B (1010). **b** The read-out process of the four-bit binary optical information. **c** Photograph of the phosphor discs array was read-out by the ultraviolet camera at room temperature under 453 K (inset i), indoor white LED light (300 lux) (inset ii) and darkness + 635 nm laser (10 mW) (inset iii). **d** The 16 results of the optical information read-out based on the 2 × 2 unit array.

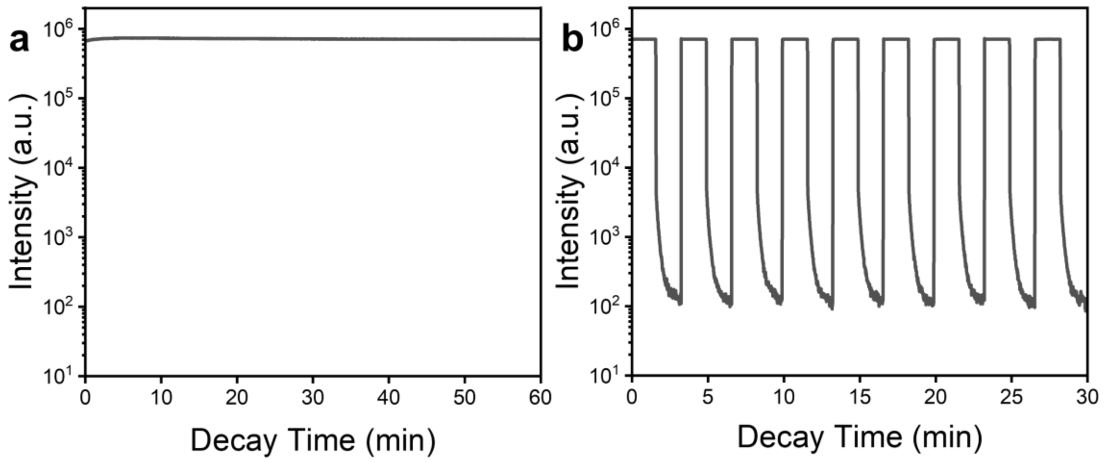

Fig. S17 a, b The decay curves monitored at 299 nm under continuous X-ray irradiation and repeated cycles of X-ray excitation.

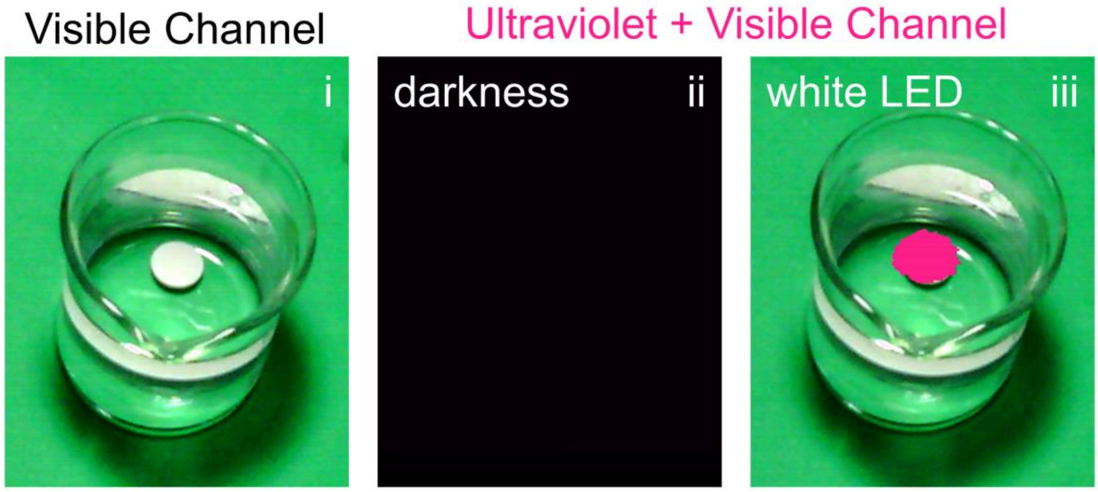

Fig. S18 Ultraviolet images of the $ScBO_3$:Bi^{3+} phosphor discs underwater recorded by the ultraviolet camera upon (**ii**) darkness and (**iii**) white LED illumination at room temperature.

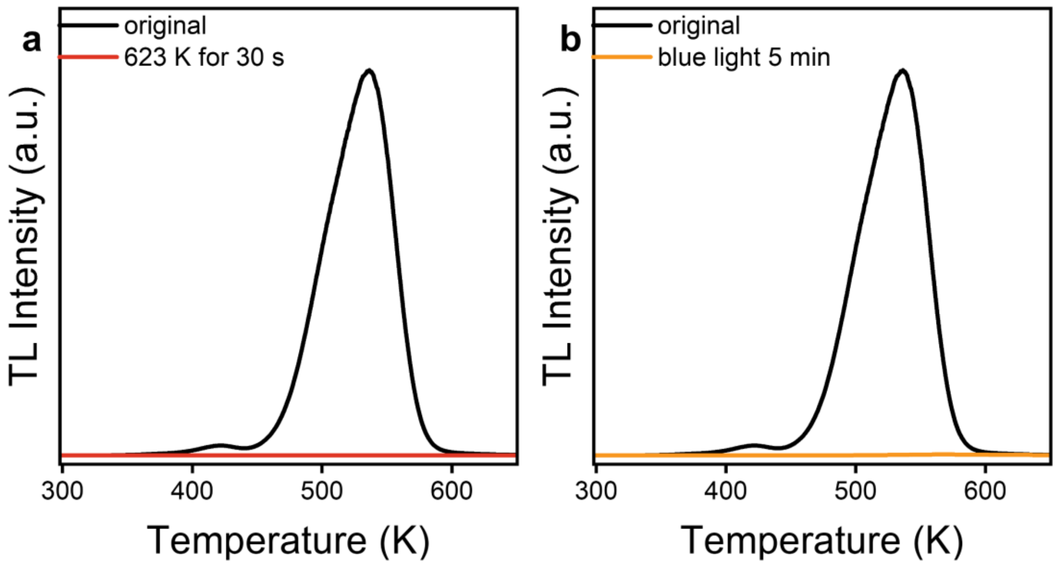

Fig. S19 a, b TL curves of the $ScBO_3$:Bi^{3+} phosphor under thermal bleaching of 623 K for 30 s and optical bleaching of the blue LED (460-465nm) for 5 min.

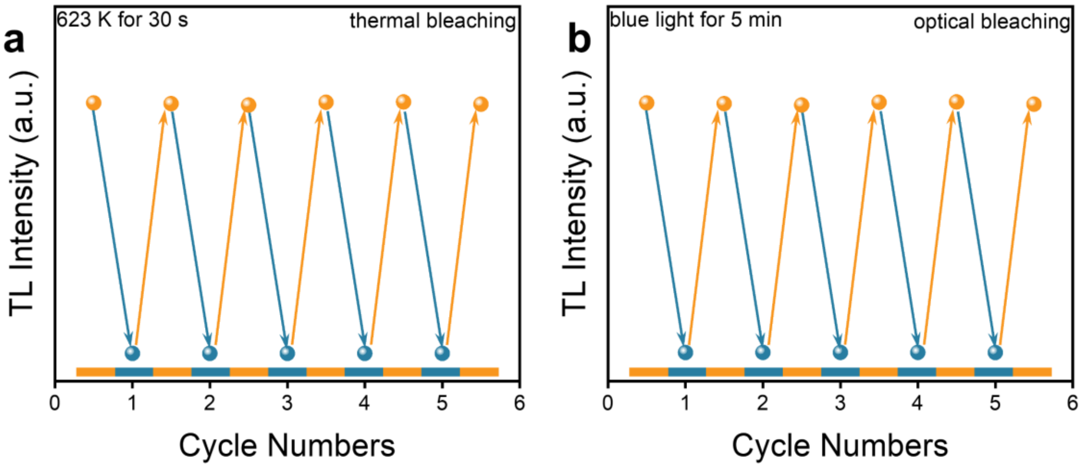

Fig. S20 Stability and repeatability of write-erase of the $ScBO_3:Bi^{3+}$ phosphor. TL intensity of the $ScBO_3:Bi^{3+}$ phosphor by alternating **a** X-ray irradiation for 25 min and thermal bleaching at 623 K for 30 s, and **b** X-ray irradiation for 25 min and optical bleaching (blue light, 460-465 nm) for 5 min as a function of cycle numbers.

Table S1 Rietveld refinement parameters of the $ScBO_3$ and $ScBO_3:Bi^{3+}$ phosphor.

Sample	$ScBO_3$	$ScBO_3:Bi^{3+}$
Space group	$R\bar{3}c$	$R\bar{3}c$
a = b (Å)	4.748	4.752
c (Å)	15.262	15.299
V(Å^3)	297.9638	299.1896
α = β (º)	90	90
γ (º)	120	120
R_{wp} and R_p	/	9.27% and 7.24%
χ^2	/	3.974
